# A global transcriptional activator involved in the iron homeostasis in cyanobacteria

**DOI:** 10.1126/sciadv.adl6428

**Published:** 2024-07-03

**Authors:** Ling-Mei Liu, Chuan-Yu Sun, Yi-Cao Xi, Xiao-Hui Lu, Cheng-Wen Yong, Shuang-Qing Li, Qiao-Wei Sun, Xin-Wei Wang, You-Zhi Mao, Weizhong Chen, Hai-Bo Jiang

**Affiliations:** ^1^ Key Laboratory of Marine Biotechnology of Zhejiang Province, School of Marine Sciences, Ningbo University, Ningbo, Zhejiang, China.; ^2^ School of Life Sciences, Central China Normal University, Wuhan, Hubei, China.; ^3^ Wuhan Frasergen Bioinformatics Co. Ltd., Wuhan, Hubei, China.; ^4^ Southern Marine Science and Engineering Guangdong Laboratory (Zhuhai), Zhuhai, Guangdong, China.

## Abstract

Cyanobacteria use a series of adaptation strategies and a complicated regulatory network to maintain intracellular iron (Fe) homeostasis. Here, a global activator named IutR has been identified through three-dimensional chromosome organization and transcriptome analysis in a model cyanobacterium *Synechocystis* sp. PCC 6803. Inactivation of all three homologous IutR-encoding genes resulted in an impaired tolerance of *Synechocystis* to Fe deficiency and loss of the responses of Fe uptake–related genes to Fe-deplete conditions. Protein-promoter interaction assays confirmed the direct binding of IutR with the promoters of genes related to Fe uptake, and chromatin immunoprecipitation sequencing analysis further revealed that in addition to Fe uptake, IutR could regulate many other physiological processes involved in intracellular Fe homeostasis. These results proved that IutR is an important transcriptional activator, which is essential for cyanobacteria to induce Fe-deficiency response genes. This study provides in-depth insights into the complicated Fe-deficient signaling network and the molecular mechanism of cyanobacteria adaptation to Fe-deficient environments.

## Teaser

The 3D chromosome organization analysis identified an important global activator for the Fe-deficiency response of cyanobacteria.

## INTRODUCTION

Cyanobacteria are ancient photosynthetic and oxygen-producing microorganisms, which play a critical role in the carbon and nitrogen cycles of the biosphere (*1*, *2*). The carbon fixed by cyanobacteria accounts for approximately 50% of the annual carbon fixation in oligotrophic open oceans (*3*, *4*). As an essential trace element for the growth and development of cyanobacteria, iron (Fe) functions as an important cofactor during many important cell processes and participates in various biochemical processes, including photosynthesis, respiration, nitrogen fixation, DNA synthesis, chromophore biosynthesis, and gene regulation (*5*). Since a large amount of Fe is required for electron transport during photosynthesis, the Fe demand of cyanobacteria is higher than that of heterotrophic organisms. For example, the intracellular Fe content of *Synechocystis* sp. PCC 6803 can reach 9.3 × 10^6^ ± 0.75 × 10^6^ atoms per cell, which is approximately 50 times higher than that of *Escherichia coli* of the same size (*6*–*8*). Although Fe is the fourth richest element in Earth’s crust, its bioavailability is extremely low. The concentration of dissolved Fe in natural seawater is generally lower than 2.5 nM (*9*–*11*). This scarcity of Fe in the natural environment greatly limits the photosynthetic activity and primary productivity of cyanobacteria (*12*, *13*).

To meet the growth requirements under Fe limitation, cyanobacteria have gradually developed unique Fe-deficiency adaptation mechanisms during a long evolutionary process, such as increasing Fe uptake and transport rates, capturing insoluble Fe by special extracellular structure, decreasing Fe demand by replacing ferredoxin with flavodoxin, and protecting the photosystems using IsiA, a chlorophyll (Chl)-binding protein (*14*–*23*). On the other hand, excess Fe in cells leads to the production of reactive oxygen species through the Fenton reaction, causing oxidative damage (*24*). Under high Fe stress conditions, cyanobacteria need to export redundant Fe using an efflux system (*22*). However, in a complicated and changeable Fe environment, regulating the intracellular Fe homeostasis only by the Fe uptake system and efflux system is not feasible. Therefore, it is important to strictly control the intracellular Fe concentration and effectively modify the regulatory network of Fe homeostasis in cyanobacteria to ensure the basic biological functions and avoid cell damage (*25*, *26*).

So far, only a few studies have focused on Fe-deficiency signal transduction and gene regulation in cyanobacteria. FurA (ferric uptake regulator) protein is the most-studied Fe-deficiency signal transduction regulator in cyanobacteria, which acts as a transcriptional repressor, combines with Fe^2+^ and inhibits the expression of genes related to Fe uptake under Fe-replete conditions, and relieves this inhibition under Fe-deplete conditions (*27*–*33*). FurA protein has been identified as a global transcriptional regulator in cyanobacteria, which not only regulates Fe homeostasis but also is involved in many other fundamental physiological processes, such as photosynthesis, respiration, heterocyst differentiation, oxidative stress defense, signal transduction, and maintaining cell morphology (*30*–*35*). Moreover, gene inactivation of FurA has been proven to be lethal to cyanobacteria (*36*). This suggests that FurA not only regulates Fe-deficiency signaling but also is essential for normal physiological processes in cyanobacteria. However, it remains unclear whether special transcriptional activators are required to induce the expression of Fe-deficiency adaptive genes after FurA dissociates from the promoters of target genes in Fe-deficient cyanobacteria. Except for the global transcription factor FurA, only a few other reports have identified the role of other factors in Fe-deficiency signal transduction in cyanobacteria. In *Synechocystis* sp. PCC 6803, a transcriptional factor SufR was reported to regulate the genes involved in the assembly of Fe-S gene clusters (*37*). PfsR, a transcriptional regulator of the TetR family, has been reported to influence the expression of genes related to Fe-deficiency adaptation, such as *fut*, *feoB*, *bfr*, *isiA*, and *furA* (*38*). Apart from these transcriptional regulatory proteins, small noncoding regulatory RNAs have also been reported to participate in the regulation of Fe homeostasis in cyanobacteria (*39*–*41*). For example, in *Synechocystis* sp. PCC 6803, IsrR (iron stress-repressed RNA) can repress the expression of *isiA* and affect the number of IsiA–photosystem I (PSI) super complex during Fe starvation through a complex network system (*40*). Similarly, IsaR1 (iron stress-activated RNA 1) also plays a pivotal role in the adaptation of photosynthetic apparatus to handle Fe deficiency in *Synechocystis* sp. PCC 6803 at three levels: (i) via posttranscriptional repression of gene expression; (ii) indirectly, via suppression of pigment biosynthesis; and (iii) through Fe-S cluster assembly (*41*). In addition to small RNAs, long-antisense RNAs (α-*furA* RNA) modulating FurA have been reported in *Anabaena* sp. strain PCC 7120, *Synechocystis* sp. PCC 6803, and *Microcystis aeruginosa* PCC 7806 (*42*, *43*). So far, apart from FurA, there is no convincing evidence of the existence of another global transcription factor that directly binds the promoter of Fe uptake–related genes and regulates Fe homeostasis in cyanobacteria.

Transcriptome analysis under Fe-deficient conditions and gene knockouts are general research approaches used to explore previously unknown transcriptional regulators involved in Fe-deficiency signal transduction. Over the past decade, the three-dimensional (3D) chromosome structure has been extensively studied in some prokaryotic species, such as *E. coli* (*44*), *Bacillus subtilis* (*45*), *Caulobacter crescentus* (*46*), *Corynebacterium glutamicum* (*47*), and *Streptomyces coelicolor* (*48*). Higher-order chromosomal structure plays a crucial role in various genome functions, such as DNA replication, DNA methylation, transcriptional regulation, and cohesin binding, and can provide a mechanical understanding of various biological processes (*49*–*52*). The frequency and intensity of local chromosomal interactions were found to be highly correlated to transcription levels (*48*). Studies showed notable changes in the interactions of chromosomal interaction domain (CID) borders under stress conditions, suggesting that these CID border genes play important roles in specific environmental adaptation. Thus, the 3D chromosome, which has not yet been reported in any cyanobacterial species to our best knowledge, may provide an effective approach to deeply explore the mechanisms of Fe-deficiency adaptation.

In this study, 3D chromosome organization and transcriptome changes in the model cyanobacterium strain *Synechocystis* sp. PCC 6803 have been studied under Fe-deficient conditions to identify the uncharacterized homologous genes involved in the regulation of Fe homeostasis. Through gene knockout, complementation analysis, yeast one-hybrid, electrophoretic mobility shift assay (EMSA), and chromatin immunoprecipitation sequencing (ChIP-seq), a global regulatory factor involved in Fe-deficiency signal transduction in cyanobacteria has been found in this study, approximately 30 years after the discovery of FurA (*27*, *53*).

## RESULTS

### *Synechocystis* sp. PCC 6803 displayed enhanced chromatin interactions under Fe deficiency

**Fig. 1.**
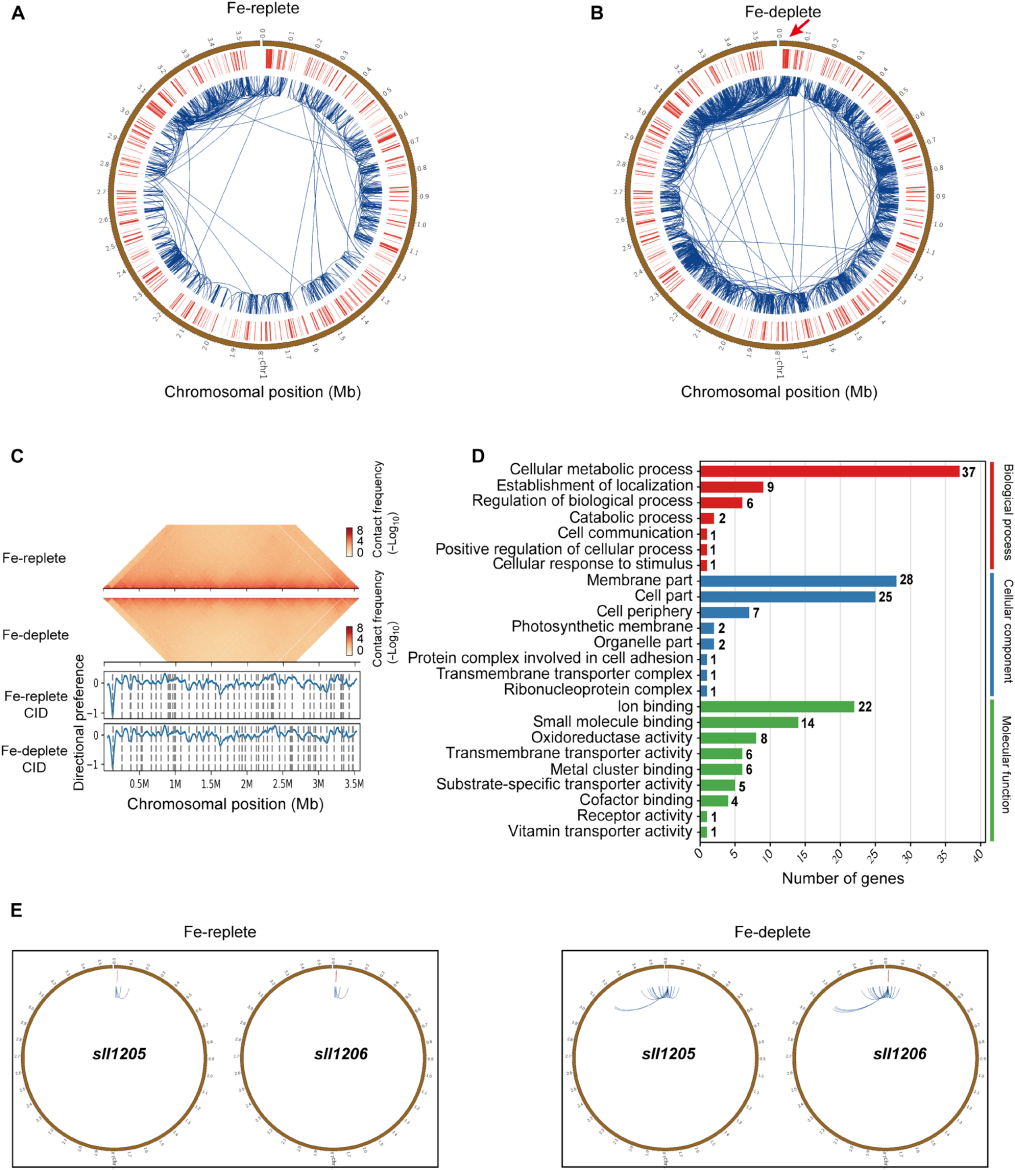
Hi-C data of *Synechocystis* sp. PCC 6803. (**A** and **B**) Circos plots representing genome-wide interactions in *Synechocystis* sp. PCC 6803 wild-type strain under Fe-replete (A) and Fe-deplete (desferrioxamine B addition, DFB) (B) conditions, respectively. (**C**) Identification of CIDs in the population of Hi-C contact-frequency matrix under Fe-replete and Fe-deplete conditions. (C) Top: Hi-C contact map of the chromosome rotated 45° clockwise with directional preference plots. Bottom: CID boundaries (vertical bars) and insulation scores. The vertical axis and the blue line in the figure represent the insulation scores, and the dashed gray lines represent CID boundaries. (**D**) Gene Ontology (GO) enrichment analysis of genes in CID boundaries specific for Fe deficiency. (**E**) Circos plots showing notably enhanced interactions in *sll1205* and *sll1206* under Fe-deplete conditions, compared to Fe-replete conditions.

To explore the changes in the spatial structure and interactions in cyanobacterial chromosomes due to Fe limitation, the 3D chromosome organization of *Synechocystis* sp. PCC 6803 under Fe-replete and Fe-deplete conditions were investigated through the high-throughput chromosome conformation capture (Hi-C) method. Since *Synechocystis* sp. PCC 6803 is polyploid and the sequences from different chromosomes are the same, their multiple chromosomes cannot be distinguished from each other. Therefore, the cyanobacterial 3D chromosome structure obtained from the Hi-C data is a representative monomeric 3D genomic model. The results showed that the chromosome architectures of *Synechocystis* sp. PCC 6803 cultured in Fe-replete and Fe-deplete conditions were similar (movies S1 and S2), indicating that the 3D chromosome structure of *Synechocystis* sp. PCC 6803 was stable. Like other bacteria (*46*, *48*), cyanobacteria have a rigorous set of high-order genomic structures maintained by nuclear-associated proteins. Therefore, epigenetic regulatory mechanisms through 3D chromosome organization also exist in prokaryotic photosynthetic bacteria, such as cyanobacteria. Notably, the chromosome of *Synechocystis* sp. PCC 6803 cultured in Fe-deplete conditions displayed a denser 3D structure than that under Fe-replete conditions (movies S1 and S2). To visualize the genomic interaction frequencies of *Synechocystis* sp. PCC 6803 under different Fe conditions, a normalized genomic contact map was prepared, in which each matrix position reflected the relative frequency of interactions between the loci on a genome scale. As shown in fig. S1A, the resulting contact maps under Fe-replete and Fe-deplete conditions exhibited strong and broad diagonals, reflecting that the local contacts resulting from the interactions among adjacent loci were frequent. The circos plot of genome-wide interactions showed that interactions under Fe-deficient conditions were obviously higher than those under Fe-replete conditions (Fig. 1, A and B). A total of 1724 and 3576 pairs of significant interaction sites were identified in *Synechocystis* sp. PCC 6803 under Fe-replete and Fe-deplete conditions, respectively, including 622 same interaction sites (fig. S1B). These results indicated that the chromosome organization of cyanobacteria underwent adaptive dynamic adjustment during Fe starvation.

CIDs are the fundamental units of the 3D chromosome structure and are related to important structural and regulatory roles in prokaryotes (*35*, *54*, *55*). CIDs refer to the contiguous genomic segments in which DNA sequences exhibit notably higher interaction frequencies than those located in different domains (*46*), similar to topologically associated domains observed previously in eukaryotic Hi-C data (*56*). The Hi-C interaction matrix in this study revealed 52 and 51 obvious CIDs in the chromosome of *Synechocystis* sp. PCC 6803 cultured under Fe-replete and Fe-deplete conditions, respectively, while only 25 intact CIDs were exactly the same (Fig. 1C and fig. S1B). A total of 257 and 273 genes were found in CID boundaries under Fe-replete and Fe-deplete conditions, respectively. Among them, only 147 intact genes were the same in *Synechocystis* sp. PCC 6803 cultured under different Fe conditions. This suggested that the CID boundaries changed substantially under different Fe conditions (fig. S1B). Gene Ontology (GO) enrichment analysis of the genes in the CID boundaries specific for Fe-deficient conditions revealed that these genes were involved in many physiological and metabolic processes, including cellular metabolic process, cell communication, ion binding, and receptor activity (Fig. 1D).

Compared with *Synechocystis* sp. PCC 6803 cultured under Fe-replete conditions, some of the most remarkable enhanced interactions were located between 0 and 0.1 Mb and 3.0 and 3.2 Mb regions of the chromosome in *Synechocystis* sp. PCC 6803 cultured under Fe-deplete conditions (Fig. 1, A and B). Many Fe uptake–related genes were located in this region, including genes related to the TonB-dependent active uptake system, such as four TonB-dependent transporter (TBDT)–encoding genes (*sll1206*, *sll1406*, *sll1409*, and *slr1490*) and TonB-encoding gene (*slr1484*), as well as some genes related to Fec family, such as FecC-encoding gene (*slr1316*) and FecB-encoding genes (*slr1492*) (Fig. 1E and fig. S2). In addition, other genes related to Fe-deficiency adaption, such as *isiB* (*sll0248*), *futC* (*sll1878*), *futA1* (*slr1295*), *feoB* (*slr1392*), and *futA2* (*slr0513*), also showed enhanced interactions under Fe-deplete conditions (fig. S2). These results indicated that the 3D chromosome structure of *Synechocystis* sp. PCC 6803 substantially remodeled with the changes in Fe concentration, especially in chromosome regions related to Fe metabolism, thereby regulating the expression of Fe-related genes to maintain intracellular Fe homeostasis. Notably, three uncharacterized potential transcription factor–encoding genes (*sll1205*, *sll1408*, and *slr1489*) were located in the region of 0 to 0.1 Mb. Among them, *sll1205* showed notably enhanced interactions under Fe starvation, indicating that it might be involved in Fe-deficiency adaption (Fig. 1E).

### Fe uptake–related genes were significantly up-regulated under Fe-deficient conditions

**Fig. 2.**
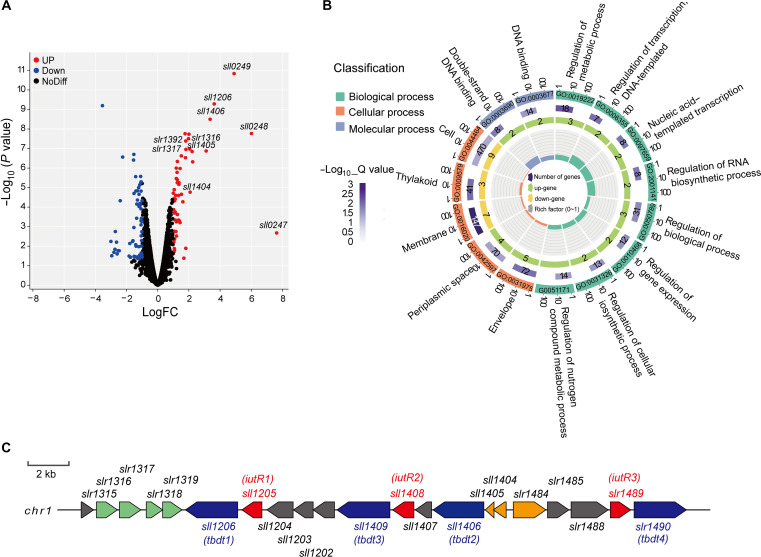
Difference in the transcriptome of *Synechocystis* sp. PCC 6803 wild-type strain under Fe-replete conditions and after 24 hours of Fe depletion by DFB addition. (**A**) Volcano plots of total gene expressions in the wild-type strain after 24 hours of Fe depletion. Blue dots represent down-regulated genes and red dots represent up-regulated genes. (**B**) GO enrichment analysis of DEGs in the wild-type strain after 24 hours of Fe depletion. (**C**) Location of *iutR* genes (*sll1205*, *sll1408*, and *slr1489*) in the genome of *Synechocystis* sp. PCC 6803.

Transcriptome analysis was performed for *Synechocystis* sp. PCC 6803 to explore the relationship between 3D chromosome structure and gene expressions under Fe-replete and Fe-deplete conditions. The results revealed that about 3.3% of the total genes showed significant differential expression between *Synechocystis* sp. PCC 6803 cultured in Fe-replete and Fe-deplete conditions. Among them, 55 genes were significantly up-regulated, while 64 genes were significantly down-regulated under Fe-deplete conditions (Fig. 2A). The significantly up-regulated genes have been summarized in Table 1. Consistent with the previous studies (*57*–*59*), *isiA* (*sll0247*) and *isiB* (*sll0248*), which are considered the marker genes for Fe deficiency, were significantly up-regulated (198.21 times and 64.72 times, respectively) under Fe-deplete conditions, as compared to Fe-replete conditions. Besides, genes related to Fe uptake were also up-regulated to some extent. For example, *tonB*, *tbdt1*, *tbdt2*, *exbB*, and *exbD* genes (related to TonB-mediated active transport) were up-regulated by 2.56, 12.34, 10.27, 4.23, and 4.15 times, respectively. Some genes related to Fe^3+^ transport (i.e., *fecC*, *fecD*, and *futA2*) and Fe^2+^ transport (i.e., *feoB*) also showed significant up-regulation (Table 1). Through GO analysis of differentially expressed genes (DEGs), 15 significantly enriched GO terms were determined (*P* < 0.05; Fig. 2B), including the regulation of RNA biosynthetic process, transcription, and gene expression.

**Table 1.** Up-regulated genes under Fe-deplete conditions in *Synechocystis* sp. PCC 6803. All the genes showed significant up-regulation under Fe-deplete conditions: fold change ≥2, *P* ≤ 0.1. NAD(P)H, reduced form of nicotinamide adenine dinucleotide; ATP, adenosine 5′-triphosphate.

Gene ID	Gene functional identification	Fold change (times)
*sll0247*	Fe stress Chl-binding protein; *isiA*	198.21
*sll0248*	Flavodoxin; *isiB*	64.72
*sll0249*	Unknown protein	29.83
*sll1206*	TonB-dependent siderophore receptor; *tbdt*	12.34
*sll1406*	TonB-dependent siderophore receptor; *tbdt*	10.27
*sll1407*	Unknown protein	8.61
*ssl0461*	Unknown protein	4.74
*sll1549*	Unknown protein	4.62
*sll1404*	Biopolymer transport ExbB protein; *exbB*	4.23
*sll1405*	Biopolymer transport ExbD protein; *exbD*	4.15
*slr1488*	ABC transporter	4.01
*slr1316*	Iron-siderophore transport system permease protein; *fecC*	3.93
*slr1317*	Iron-siderophore transport system permease protein; *fecD*	3.51
*sll0614*	Unknown protein	3.49
*slr1392*	Ferrous iron transport protein B; *feoB*	3.48
*slr1185*	Cytochrome b6-f complex iron-sulfur subunit; *petC*	3.44
*ssr2333*	Ferrous iron transport protein A; *feoA*	3.38
*sll0864*	Unknown protein	3.20
*sll1451*	Nitrate/nitrite transport system permease protein; *nrtB*	3.06
*sll1154*	Quinolone resistance protein; *norA*	2.86
*slr1186*	Unknown protein	2.71
*sll1202*	Unknown protein; homology with *fhuD*	2.70
*slr1293*	Unknown protein	2.62
*sll1452*	Nitrate/nitrite transport system ATP-binding protein; *nrtC*	2.58
*slr1484*	TonB protein; *tonB*	2.56
*slr1485*	Putative phosphatidylinositol phosphate kinase	2.53
*slr1919*	Unknown protein	2.53
*slr0228*	Cell division protease FtsH; *ftsH*	2.40
*sll1550*	Porin	2.40
*sll0141*	Efflux resistance-nodulation-cell division transporter periplasmic adaptor subunit	2.39
*slr0513*	Iron(III) transport system periplasmic iron-binding protein; *futA2*	2.39
*slr0119*	Membrane protein; *brkB*	2.32
*sll1483*	Transforming growth factor–induced protein	2.31
*sll1205*	Regulatory protein; *iutR1*	2.30
*sll2012*	RNA polymerase nonessential primary-like sigma factor; *rpoD*	2.29
*sll1450*	Nitrate/nitrite transport system substrate-binding protein; *nrtA*	2.28
*slr0611*	All-trans-nonaprenyl-diphosphate synthase; *sds*	2.25
*slr0118*	Phosphomethylpyrimidine synthase; *thiC*	2.23
*slr1489*	Regulatory protein; *iutR3*	2.17
*slr1501*	Unknown protein	2.16
*sll1408*	Regulatory protein; *iutR2*	2.11
*sll7086*	Unknown protein	2.11
*slr0041*	Bicarbonate transport system permease protein; *cmpB*	2.10
*ssr6030*	Unknown protein	2.10
*slr1291*	NAD(P)H-quinone oxidoreductase subunit 4; *ndhD2*	2.09
*slr1544*	Unknown protein	2.07
*sll8027*	Unknown protein	2.07
*ssl5114*	Unknown protein	2.06
*sll7089*	CRISPR-associated protein; *cmr3*	2.05
*slr2077*	Unknown protein	2.03
*ssl0453*	Phycobilisome degradation protein; *nblA*	2.00

Consistent with the results of 3D chromosome, the genes in the 0- to 0.1-Mb region with enhanced interactions, such as *iutR1* (*sll1205*), *tbdt1* (*sll1206*), *fecC* (*slr1316*), *isiB* (*sll0248)*, *feoB* (*slr1392*), *futA2* (*slr0513*), and *tonB* (*slr1484*), were significantly up-regulated under Fe-deplete conditions (Fig. 2C and Table 1). Among them, *sll1205*, *sll1408*, and *slr1489*, which are highly homologous and annotated as transcription factors, were significantly up-regulated by 2.3, 2.11, and 2.27 times, respectively, under Fe-deplete conditions, as compared to Fe-replete conditions (Table 1). These three genes were located upstream of the genes that encode three outer membrane proteins (TBDT) in the TonB-dependent active uptake system, respectively [Kyoto Encyclopedia of Genes and Genomes (KEGG), www.kegg.jp/] (Fig. 2C). Protein structure prediction using AlphaFold2 revealed that all the three proteins (Sll1205, Sll1408, and Slr1489) contained the typical transcription factor domain helix-turn-helix and belonged to the AraC family of transcription factors (fig. S3). This indicates that these three proteins are typical transcriptional regulators. These three genes were annotated as *pchR* in cyanobacteria based on the homology to PchR (pyochelin transport regulator), a transcriptional regulator of endogenous siderophores in Gram-negative bacterium such as *Pseudomonas aeruginosa* (*60*, *61*). Since *Synechocystis* sp. PCC 6803 cannot secrete endogenous siderophores such as pyochelin, to intuitively reflect the role of these genes in cyanobacteria, we renamed them as *iutR1*, *iutR2*, and *iutR3* (iron uptake–related transcriptional regulator), respectively, for further investigation.

### The *iutR* triplex mutant showed higher low-Fe sensitivity than the wild-type strain

**Fig. 3.**
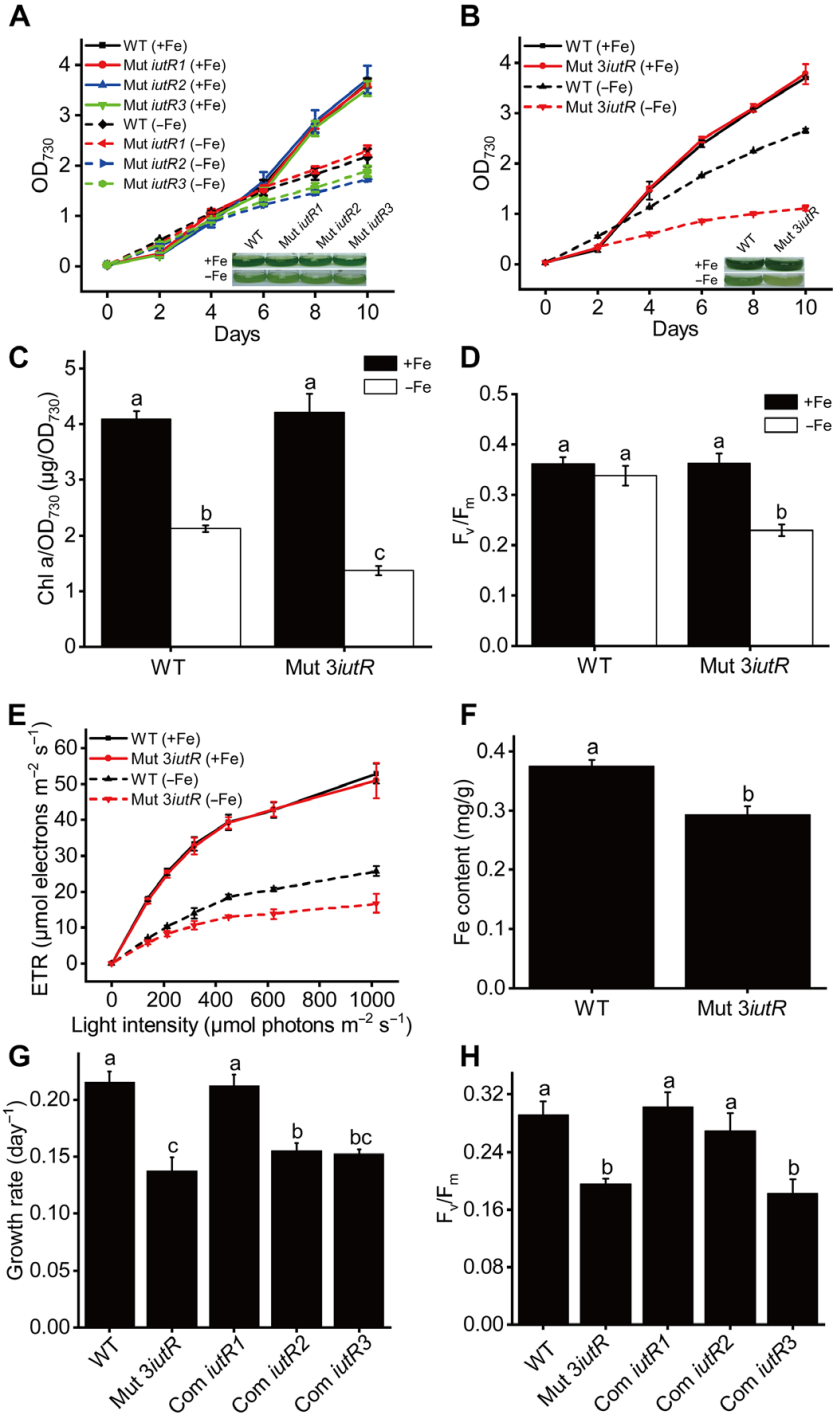
Physiological phenotypes of the wild-type (WT) strain, *iutR* mutants, and complementation strains grown under Fe-replete (+Fe) and Fe-deplete (−Fe) conditions. (**A**) Growth curves and photographs of wild type (WT), *iutR1* mutant, *iutR2* mutant, and *iutR3* mutant under +Fe and −Fe conditions. (**B** to **F**) Growth curves and photographs (B), Chl a content (C), F_v_/F_m_ (D), electron transfer rate (ETR) (E), and Fe content (F) in WT and *IutR* triplex mutant under +Fe and −Fe conditions. (**G**) Growth rate of WT, *iutR* triplex mutant, Com *iutR1*, Com *iutR2*, and Com *iutR3* on the eighth day under −Fe conditions. (**H**) F_v_/F_m_ of WT, *iutR* triplex mutant, Com *iutR1*, Com *iutR2*, and Com *iutR3* on the eighth day under −Fe conditions. The final Fe concentrations were 21.4 μM and 0 nM for the Fe-replete and Fe-deplete cultures, respectively. The inset images in (A) and (B) show the photographs of the WT strain and the mutant strains on the fourth day under +Fe and −Fe conditions. The Chl a content, F_v_/F_m_, and ETR were all measured on the fourth day.

To verify whether IutR proteins are required for the regulation of Fe homeostasis in *Synechocystis* sp. PCC 6803, IutR-encoding genes *sll1205*, *sll1408*, and *slr1489* were knocked out by insertion of the kanamycin-resistant (C.K2), erythromycin-resistant (C.CE2), and gentamicin-resistant (Gm) cassette fragments, respectively (fig. S4A). As confirmed by polymerase chain reaction (PCR), the resulting mutant strains were entirely segregated (fig. S4D). Growth curve assay showed that inactivation of the single *iutR* gene had an inconspicuous influence on the growth of *Synechocystis* sp. PCC 6803 under both Fe-replete and Fe-deplete conditions (Fig. 3A). This phenomenon can be explained by the fact that three IutRs are highly homologous, and the functional inactivation of one IutR protein can be compensated by the other two redundant IutR proteins. On the basis of this fact, all three IutR-encoding genes were knocked out to obtain an *IutR* triplex mutant (fig. S4, B and D). The growth curve assay showed that the inactivation of all three *iutRs* had no impact on the growth of cyanobacteria under Fe-replete conditions (Fig. 3B). However, the *iutR* triplex mutant grew much slower than the wild-type strain under Fe-deplete conditions (Fig. 3B). The changes in the Chl a content were consistent with the growth curve (Fig. 3C), indicating that the *iutR* triplex mutant was more sensitive to Fe deficiency than the wild-type strain. Moreover, the maximum photochemical efficiency of PSII (F_v_/F_m_) and electron transfer rate (ETR) of the *iutR* triplex mutant were lower than those of the wild-type strain under Fe-deplete conditions (Fig. 3, D and E). Furthermore, the intracellular Fe content of the *iutR* triplex mutant was less than that of the wild-type strain (Fig. 3F). This indicated the involvement of IutR in Fe-deficiency adaption in *Synechocystis* sp. PCC 6803.

To exclude the false-positive mutant strain resulting from the second point mutation, the three *iutR* genes were complemented in the *iutR* triplex mutant separately (named Com *iutR1*, Com *iutR2*, and Com *iutR3*, respectively) (fig. S4, C and D). The growth rates showed that the complementation of *iutR1* (*sll1205*) could effectively recover the low-Fe sensitive phenotype of the *iutR* triplex mutant, while the complementation of *iutR2* (*sll1408*) and *iutR3* (*slr1489*) could only partially recover the Fe sensitive phenotype (Fig. 3G). The results of F_v_/F_m_ were consistent with the observed growth rates (Fig. 3H). These results further confirmed the important role of IutRs in the adaptation of cyanobacteria to Fe deficiency. Furthermore, the findings suggested that IutR1 might show higher biological activity in cyanobacterial Fe-deficiency adaptation than the other two IutR proteins.

### Knockout of *iutRs* inhibited the induction of Fe uptake–related genes under Fe deficiency

**Fig. 4.**
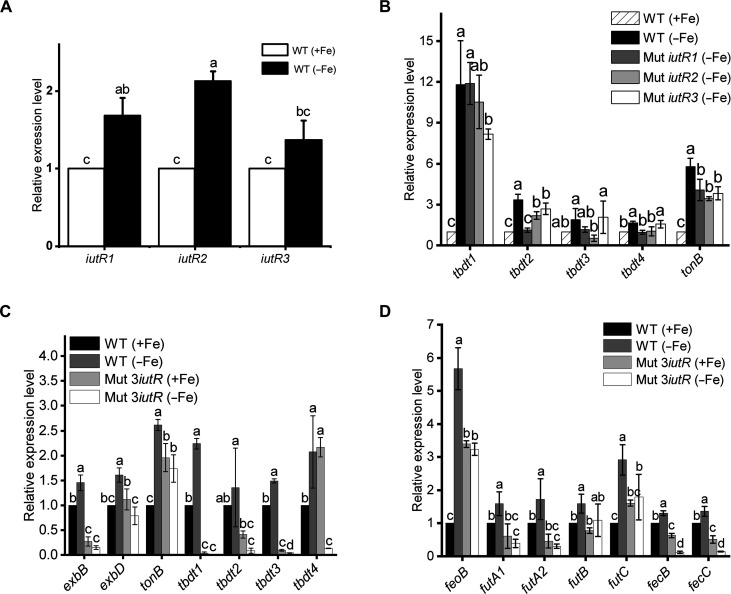
Transcription analysis of Fe uptake–related genes in *Synechocystis* sp. PCC 6803 by RT-qPCR. (**A**) Transcription levels of *iutR1*, *iutR2*, and *iutR3* in the WT strain cultured in Fe-replete (+Fe) and Fe-deplete (−Fe) BG11 medium for 24 hours. (**B**) Transcription levels of Fe uptake–related genes in the WT strain and three *iutR* single mutants. (**C** and **D**) Transcription levels of Fe uptake–related genes, including TBDT-encoding genes (C) and other Fe transport system genes (D) in the WT strain and *iutR* triplex mutant. The final Fe concentrations were 21.4 μM and 0 nM for the Fe-replete and Fe-deplete cultures, respectively.

It is well known that many Fe uptake–related genes show up-regulated expression under Fe-deficient conditions. To investigate whether the knockout of IutRs influenced the expression of Fe uptake–related genes, reverse transcription quantitative real-time PCR (RT-qPCR) was conducted. The results showed that the expressions of genes related to TonB-dependent Fe transport system, such as *tbdt1* (*sll1206*), *tbdt2* (*sll1406*), *tbdt3* (*sll1409*), *tbdt4* (*slr1490*), and *tonb* (*slr1484*), as well as *iutR1*, *iutR2*, and *iutR3* in the wild-type strain were significantly up-regulated after 24 hours of growth in Fe-deplete medium (Fig. 4, A and B). These results were consistent with the abovementioned results of transcriptome analysis. By contrast, the up-regulation of these Fe uptake genes in the three *iutR* single mutants was obviously less compared to the up-regulation in the wild-type strain under Fe-deplete conditions (Fig. 4B). In the *iutR* triplex mutant, however, these genes, including TBDT-encoding genes, Fe^2+^ transporter FeoB–encoding gene (*feoB*), Fe^3+^ transport system–encoding genes (*futA1*, *futA2*, *futB*, and *futC*), and Fec family–encoding genes (*fecB* and *fecC*) were not up-regulated at all, and some of them were even down-regulated under Fe-deplete conditions. This indicated that knockout of all three *iutR* genes seriously inhibited the expression of these Fe uptake–related genes (Fig. 4, C and D). These results revealed that IutR acted as a transcriptional activator to regulate the expression of diverse Fe uptake pathways, and the inactivation of all IutRs led to the loss of response to Fe-deficiency signals in *Synechocystis* sp. PCC 6803.

### IutRs bind directly to the promoters of TBDT-encoding genes

A protein-protein interaction research through yeast two-hybrid assays revealed that there were no interactions between the three IutR proteins (fig. S5A). Considering the phenotypes of three *iutR* single mutants and three complementation strains, the possibility of the three IutR proteins forming heteromultimer to perform their functions was ruled out. It suggested that the three IutR homologs are likely to perform functions individually.

Since IutRs are predicted as typical transcriptional regulators by protein structure prediction (fig. S3) and located upstream of the *tbdt* genes in the genome (Fig. 2C), we hypothesize that IutRs might directly bind with the *tbdt* promoters to regulate gene expression. Thus, EMSA was conducted and the results showed that IutR1 interacted with the promoters of *tbdt1*, *tbdt3*, and *tbdt4*, while IutR2 interacted with the promoters of *tbdt1* and *tbdt4*; IutR3 had interaction with the promoter of *tbdt4* (Table 2). The interactions between IutR1 and *tbdt1*, IutR2 and *tbdt1*, and IutR3 and *tbdt4* were further verified by the yeast one-hybrid assay (Table 2 and fig. S5B). These results demonstrated that transcription factor IutR could regulate the expression of TBDT-encoding genes by directly binding with the promoters of target genes.

**Table 2.** The interactions of IutRs and TBDT promoters in *Synechocystis* sp. PCC 6803. “+” means that there is interaction, “−” means that there is no interaction, and “N” means undetected.

Interaction of IutR and TBDT	EMSA assay	Yeast one-hybrid assays
IutR1 and TBDT1	+	+
IutR1 and TBDT3	+	N
IutR1 and TBDT4	+	N
IutR2 and TBDT1	+	+
IutR2 and TBDT3	−	N
IutR2 and TBDT4	+	N
IutR3 and TBDT1	−	N
IutR3 and TBDT3	−	N
IutR3 and TBDT4	+	+

### IutR functions as a global transcriptional regulator in cyanobacterial Fe-deficiency adaption

**Fig. 5.**
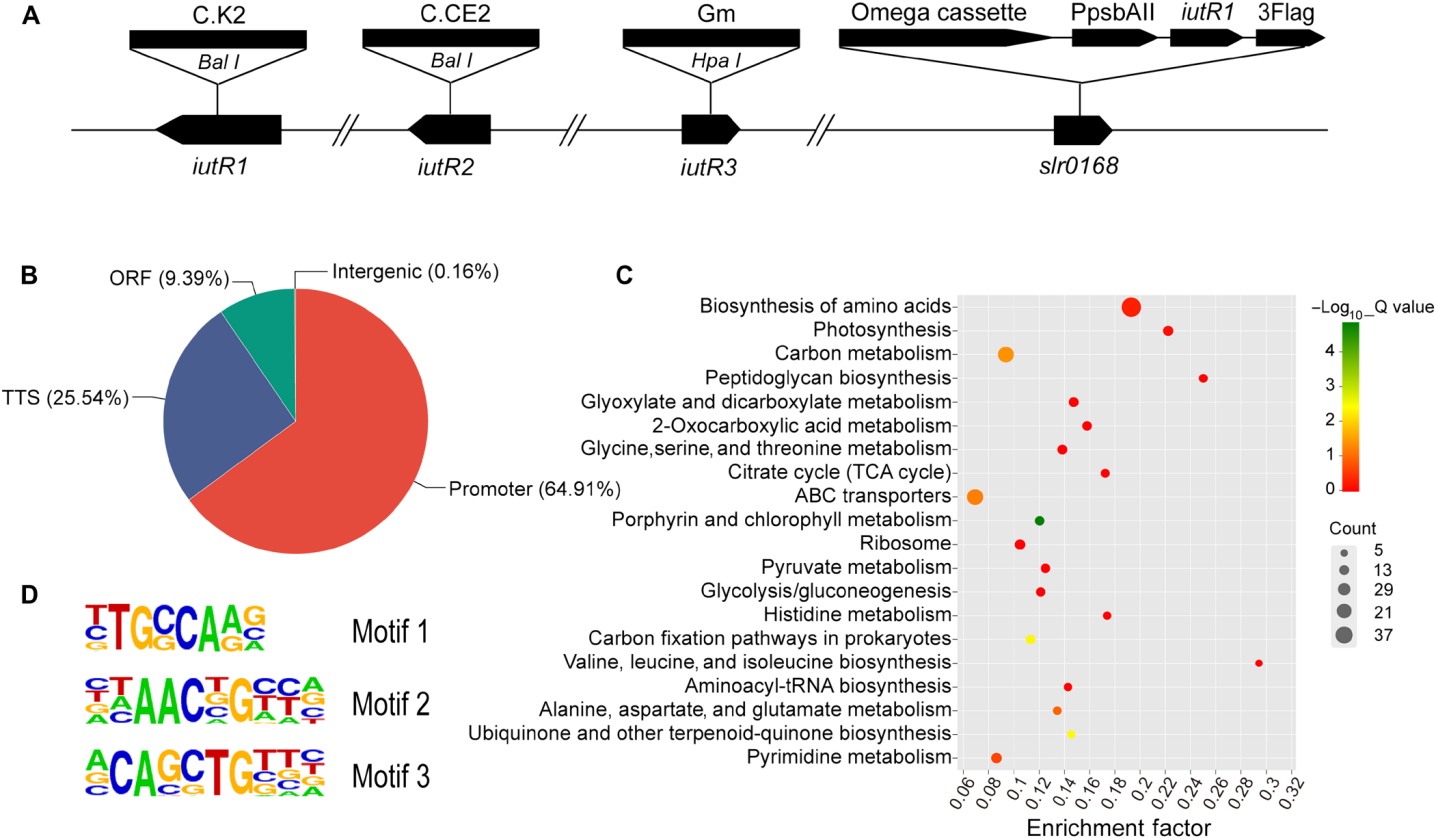
ChIP-seq assay showing the genes regulated by IutR1. (**A**) Sketch map of Com-*iutR1*–3× Flag strain. (**B**) Distribution of IutR1 binding sites across different genomic functional regions. TSS and TTS indicate transcription start sites and transcription termination sites, respectively. The percentage numbers represent the ratios of binding sites in each region. (**C**) KEGG pathway enrichment analysis of genes that may be regulated by IutR1. Numbers in the *x* axis present the enrichment of gene products. (**D**) The three potential binding motifs of IutR1.

ChIP-seq assay was performed to investigate potential target genes regulated by IutR in the genome of *Synechocystis* sp. PCC 6803. Since *iutR1* showed the best complementation among three *iutR*s during *iutR* complementation assays, it may have a more important role than the other two *iutR*s in cyanobacterial low Fe adaptation. Therefore, IutR1 (Sll1205) fused with 3× Flag tag was introduced into the *iutR* triplex mutant strain as shown in Fig. 5A, and the resulting complementation strain was named as Com-*iutR1*–3× Flag strain Western blot assay confirmed that the 3× Flag-labeled IutR1 was efficiently expressed in the complementation strain (fig. S6). After 24-hour growth under Fe-deficient conditions, Com-*iutR1*–3× Flag strain was harvested for ChIP-seq assay. The DNA fragments were immunoprecipitated and the peak sequences of 150– to 450–base pair (bp) length were filtered for further bioinformatic analysis. Around 65% of the peaks corresponded to promoter transcription start site (TSS), indicating that the ChIP-seq assay was well conducted (Fig. 5B). Three most likely motifs recognized by IutR1 were determined through bioinformatic methods (Fig. 5D). KEGG pathway enrichment analysis was performed to classify the genes whose TSS sequence were baited by IutR1. The results revealed that these genes were involved in 50 metabolic pathways. Among these pathways, the top three were amino acid biosynthesis, ATP-binding cassette (ABC) transporters, and carbon metabolism (Fig. 5C).

As shown in Table 3, the genes regulated by IutR1 were classified into seven types of functions, including Fe uptake, metal ions transfer, sugar transport, amino acid transport, phosphate/nitrate transport, adenosine 5′-triphosphate (ATP)–binding, and unknown function. As expected, TBDT-encoding genes identified by yeast one-hybrid assay and EMSA were also detected during the ChIP-seq assay (Table 3). Besides, many other genes involved in Fe-deficiency adaptation were baited by IutR1, such as the genes related to Fe^3+^ uptake transporters FutABC, Fec family system, as well as *isiB* and *ftr1* genes*.* FurA may also be regulated by IutR1. These results indicated that IutR1 could regulate various genes and pathways involved in Fe-deficiency adaption in addition to the TonB-dependent active uptake system. Moreover, motif-1 and motif-2 in the promoter region showed the highest occurrence frequency among the Fe uptake–related genes (Table 3), suggesting that the interactions of IutR1 with these gene promoters may depend on these two motifs mainly.

**Table 3.** Genes that may be regulated by IutR1 as analyzed by ChIP-seq assay.

Function	Gene ID, gene annotation, and function	Promoter motif
Fe uptake–related	*slr0964*, FTR1, iron permease	Motif-1, -2, -3
	*sll1206*, TBDT, TonB-dependent transporter	Motif-1, -2, -3
	*sll1406*, TBDT, TonB-dependent transporter	Motif-1
	*slr1484*, TonB	Motif-2
	*sll0188*, Sll0188, unknown protein	Motif-2, -3
	*slr1841*, Slr1841, probable porin	Motif-2
	*sll1878*, FutC, ABC transporter	Motif-2
	*slr1485*, Slr1485, salt-induced periplasmic protein	Motif-1, -2
	*sll0248*, IsiB, flavodoxin	Motif-1
	*sll0567*, FurA, ferric uptake regulation protein	Motif-1
	*slr1738*, PerR, transcription regulator Fur family	Motif-2
	*slr1492*, FecB, Fe^3+^ dicitrate transport system substrate- binding protein	Motif-2
	*slr1317*, FecD, ABC-type Fe^3+^ dicitrate transport system permease protein	Motif-2
	*slr0513*, FutA2, periplasmic iron-containing protein FutA2	Others
	*slr1295*, FutA1, periplasmic binding protein-dependent ABC transporter	Others
	*slr1318*, FecE, Fe^3+^ dicitrate transport system ATP-binding protein	Motif-3
	*sll0240*, Sll0240, ABC transporter ATP-binding protein	Motif-1, -2
	*sll0238*, Sll0238, Fe^3+^ transport system permease protein	Motif-1, -3
	*slr0327*, FutB, periplasmic binding protein-dependent ABC transporter	Motif-1, -3
Other metal transfer–related	*sll0384*, CbiQ, cobalt/nickel transport system permease protein	Others
	*sll0382*, Slr0382, nickel transport protein	Motif-1
	*sll1598*, MntC, Mn transporter MntC	Others
Sugar transport	*slr1202*, Slr1202, permease protein of sugar ABC transporter	Motif-2, -3
	*slr1723*, Slr1723, permease protein of sugar ABC transporter	Motif-1, -2
	*slr0044*, CmpD, bicarbonate transport system ATP-binding protein	Motif-1, -2
	*slr0043*, CmpC, bicarbonate transport system ATP-binding protein	Others
	*slr0531*, GgtD, glucosylglycerol transport system per mease protein	Motif-2
	*slr0977*, RfbA, ABC transporter, permease component	Others
	*slr2108*, RfbB, polysaccharide ABC transporter ATP binding	Others
Amino acid transport–related	*slr0467*, Slr0467, ABC transporter for natural amino acids	Motif-1, -2
	*sll0764*, UrtD, urea transport system ATP-binding protein	Motif-1
	*slr0559*, Slr0559, ABC transporter for natural amino acids	Motif-1
	*slr0949*, NatD, ABC-type Nat permease for neutral amino acids	Motif-4
	*sll1041*, CysA, similar to sulfate transport ATP-binding protein	Motif-2
Phosphate transport– and nitrate transport–related	*sll0540*, PstS, phosphate-binding protein PstS homolog	Motif-2, -3
	*sll0683*, PstB, phosphate transport ATP-binding protein PstB	Motif-2
	*sll0681*, PstC, phosphate transport system permease protein PstC	Motif-2
	*sll1452*, NrtC, nitrate/nitrite transport system ATP-binding protein	Motif-2
Associated with ATP	*slr0251*, Slr0251, ATP-binding protein of ABC transporter	Motif-2, -3
	*slr0354*, Slr0354, ATP-binding protein of ABC transporter	Others
	*sll1623*, NdhM, ABC transporter ATP-binding protein	Motif-2
Unknown function	*slr1365*, Slr1365, BioY hypothetical protein	Motif-1, -3
	*sll1002*, YCF22, hypothetical protein YCF22	Motif-1, -2
	*slr1045*, YCF63, hypothetical protein YCF63	Others

To validate whether these potential targets identified by ChIP-seq assay can be regulated by IutR1, we selected more Fe homeostasis–related genes such as *slr0696* (*ftr1*), *slr1485*, *sll0248* (*isiB*), *sll0567* (*furA*), and *slr0738* (*perR*) and compared their expression levels in *iutR1* mutant and wild-type strains with RT-qPCR methods. The expression levels of these genes in the wild-type strain were significantly up-regulated during Fe deficiency. However, these genes in the *iutR1* mutant could not be up-regulated or even down-regulated under Fe-deficient conditions (fig. S8, A to E). These results further indicated that these genes were regulated by *iutR1*. In addition, the EMSA assay further proved that IutR1 can bind to the promoters of *furA* and *perR* (fig. S8, F and G). These results strongly suggested that IutR is a global transcriptional regulator and plays an important role in the regulatory network of Fe homeostasis in cyanobacteria.

## DISCUSSION

### Changing the 3D chromosome structure is an important strategy for Fe-deficiency adaption

At present, the response of cyanobacteria to Fe-deficient environments has been extensively studied at the transcriptional and protein levels (*62*–*65*), but not at the genome level. Chromosomal DNA is compacted thousands of times to fit in the confined space of cells. Unlike the eukaryotic nucleus, which is covered by a nuclear membrane, the chromosomal DNA of prokaryotes forms a constrained non-membrane structure, called the nucleoid, which is a dynamic macromolecular complex holding the hereditary substance and its associated proteins (*44*). Research on bacterial chromosome folding began in the 1970s. However, the absence of powerful tools hindered the dissection of high-order chromosomal organization. In recent years, the emerging chromosome 3D organization detection techniques allowed a more systematic and quantitative characterization of genome topology and organization. These techniques demonstrated the important role of bacterial chromosome organization in biological processes such as gene transcription, replication, repair, and regulation, thereby affecting the assessment of cell fate and phenotypes (*66*, *67*). According to the environment, the 3D chromosome structure undergoes dynamic variations, which, in turn, affect the type and intensity of gene expression (*48*).

To the best of our knowledge, there are no reports on the 3D chromosome organization of cyanobacteria so far. This study revealed that the cyanobacterial cells exhibited a denser chromatin structure and showed notably enhanced interactions across the whole chromosome under Fe-deficient conditions. This indicated that cyanobacteria could adapt to Fe-deficient environments by regulating gene expression through changes in the 3D structure of chromosomes. Among the enhanced interactions under Fe-deficient conditions, one of the most notably enhanced interactions was in the 0.0- to 0.1-Mb genomic region, which contained most of the known genes related to Fe uptake (Fig. 1B). Consistent with the result of 3D chromosome organization, the transcriptome results revealed that the genes related to Fe uptake and transport in the 0.0- to 0.1-Mb region were also significantly up-regulated under Fe-deficient conditions. Previous studies have shown that CID boundaries have higher gene density and expression levels compared to the interior (*48*, *68*). GO enrichment analysis of specific CID boundary genes under Fe deficiency revealed that these genes were involved in various functions, including cell communication, protein complexes involved in cell adhesion, transmembrane transporter complexes, ion binding, metal localization, and cell response to stimuli. This suggests that these processes are important for cyanobacterial adaption to Fe-deficient environments. Furthermore, these results indicate that it is important to regulate the gene expression at both genomic and transcription levels for Fe-deficiency adaption in cyanobacteria. The study also suggests that the analysis of the 3D chromosomal DNA structure is beneficial to understanding the adaption strategy of cyanobacteria at the genome level during Fe starvation, which cannot be achieved through transcriptome analysis. Combining 3D chromosomal organization with transcriptome analysis under different environmental conditions is an efficient strategy to explore the genes related to environmental adaptation.

### IutR is a global transcriptional activator that is essential for the Fe-deficiency signaling

Fe homeostasis is important for cyanobacteria to cope with complex and changing environments. Under Fe-replete conditions, TonB-dependent active transport in *Synechocystis* sp. PCC 6803 is either unexpressed or lowly expressed, and Fe is obtained mainly through porin-mediated passive diffusion (*17*). Under Fe-deplete conditions, these Fe active transport pathways are up-regulated to promote Fe acquisition. Transcriptional factors serve as an important link between the signaling of Fe deficiency and the genes involved in Fe uptake, storage, and efflux in cyanobacteria. Previous reports have shown that the Fe homeostasis in cyanobacteria is mainly regulated by the transcriptional repressor FurA, which inhibits the expression of Fe uptake–related genes by binding with their promoters (*27*–*36*, *69*).

In this study, three transcription factors (IutR1, IutR2, and IutR3) were identified by chromosome 3D organization and transcriptome analysis. Fe uptake–related genes could not be up-regulated in the *iutR* triplex mutant under Fe-deplete conditions (Fig. 4, C and D), which provides strong evidence that IutR is an important transcriptional activator for the regulation of Fe homeostasis in cyanobacteria. The induction of Fe uptake–related genes was also inhibited in the three *iutR* single mutants (Fig. 4B). The functions of the three IutRs were found to be redundant due to the high similarity in their amino acid sequences. Thus, the Fe uptake–related genes in *iutR* single mutants could still be up-regulated under Fe deficiency. However, the amplitude of gene up-regulation in *iutR* single mutants was substantially lower than that in the wild-type strain. Knockout of all IutRs resulted in the inability of Fe uptake–related genes to respond to Fe deficiency at all, and the *iutR* triplex mutant lost the capability of Fe-deficiency signal transduction.

EMSA, yeast one-hybrid, and ChIP-seq assays further confirmed that IutR is a global regulator and performs its function through direct binding with the promoters of target genes. Notably, the inactivation of IutR severely affected the expression of genes related to Fe^3+^ uptake (such as TBDT, Fec, and Fut system genes), while the genes related to Fe^2+^ uptake, such as *feoB*, were less affected (Fig. 4, C and D). This suggests that IutR is possibly more inclined to sense signals of Fe^3+^. Given the facts that Fe^3+^ catalyzes the Fenton reaction and causes oxidative stress in cells, as well as that the expression of the key regulator PerR in oxidative stress can be regulated by IutR1, we speculate that IutR also participates in the regulation of oxidative stress in cyanobacteria. The specific mechanism of the sensing of Fe^3+^ and Fe^2+^ signals by IutR needs to be studied further. In addition, the *iutR1* complemented strain had a good compensatory effect and its physiological phenotype was the same as the phenotype of wild-type strain. On the other hand, *iutR2* and *iutR3* only partially restored the Fe-deficiency–sensitive phenotype of the *iutR* triplex mutant (Fig. 3, G and H). EMSA and yeast one-hybrid assays also showed that the binding sites of the three IutRs were different (fig. S5). These results indicate that although the three IutRs are highly homologous, they regulate different Fe uptake–related genes separately and may perform partly different functions in cyanobacteria.

### IutR is likely to regulate Fe homeostasis in cyanobacteria together with FurA

As a global transcriptional regulator, FurA is widely distributed in cyanobacteria. When the environmental Fe is insufficient, FurA dissociates from Fe ions and relieves the repression of the target genes (*70*, *71*). Although FurA was identified approximately 30 years ago, the role of other global transcription factors in the regulation of Fe uptake was not clear. A previous study has confirmed that *furA* is an essential gene in cyanobacteria, and knockout of *furA* would lead to the death of cyanobacteria (*36*). This suggests that, in addition to the regulation of Fe homeostasis, FurA also participates in other essential physiological activities in cyanobacteria. On the contrary, the knockout of IutR-encoding genes in this study did not affect the physiological state of cyanobacteria in Fe-replete environments (Fig. 3, A and B). The *iutR* triple mutant only influenced the expression of the Fe-deficiency adaptation genes under Fe-deficient conditions. This indicated that the function of the *iutR* gene is more specific in the regulation of Fe homeostasis in cyanobacteria, as compared to FurA.

**Fig. 6.**
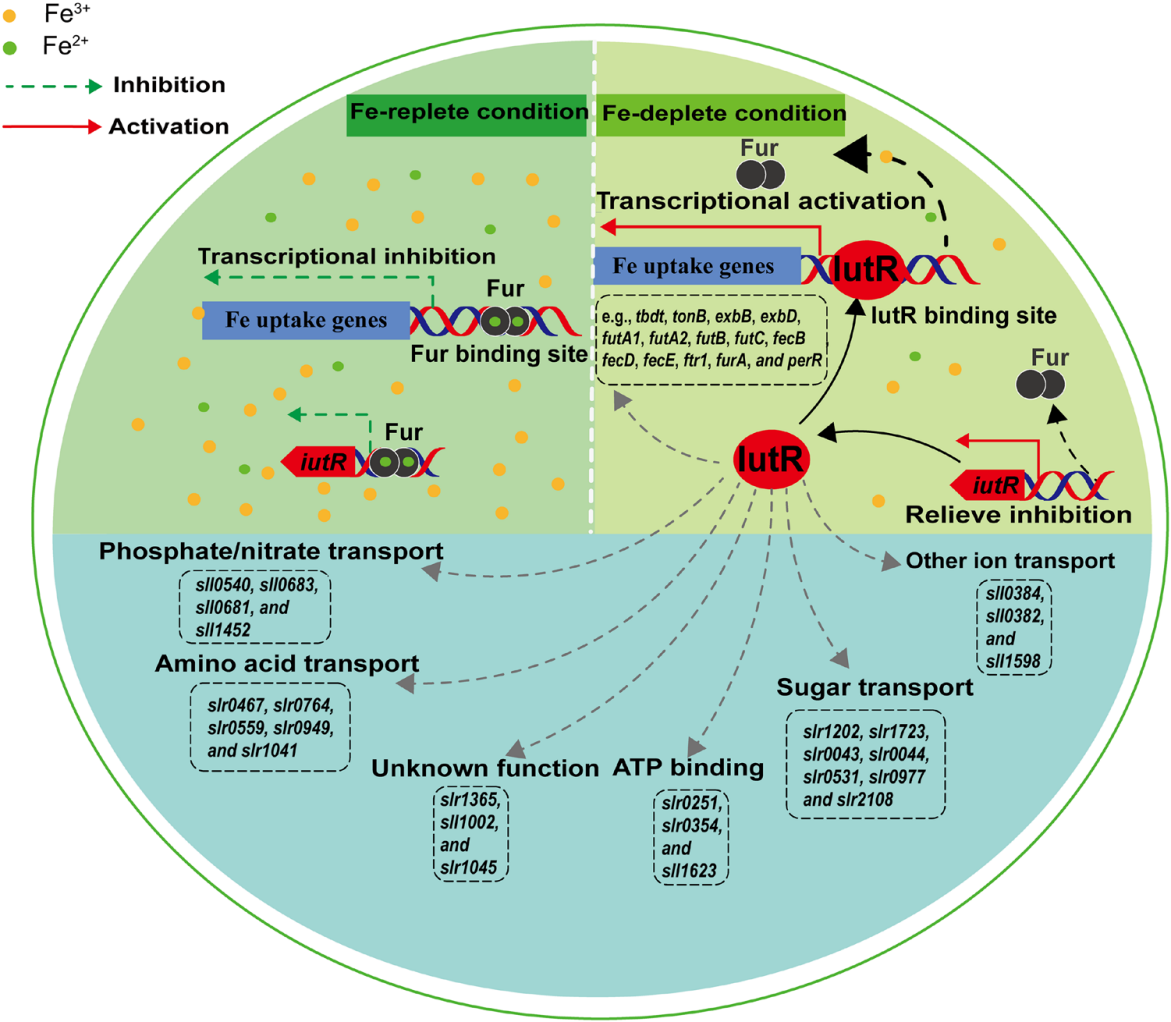
The possible working mechanism of IutR and FurA in cyanobacterial Fe homeostasis. Dashed gray arrows represent possible interactions of IutR.

Furthermore, the possible working mechanism of FurA and IutR in cyanobacterial Fe homeostasis was speculated. As shown in Fig. 6, when Fe in the environments was sufficient, FurA formed a complex with Fe^2+^ and inhibited the expression of Fe uptake–related genes. When cells were facing Fe deficiency, FurA alleviated the repression of gene expressions, and IutRs bound to the promoter and activated the expression of Fe uptake–related genes and the pathways involved in Fe-deficiency adaptation (Fig. 6). Detailed mechanisms of the joint regulation by FurA and IutRs need be clarified in the future.

### IutR is mainly distributed in cyanobacteria species in habitats with varying Fe concentration

**Fig. 7.**
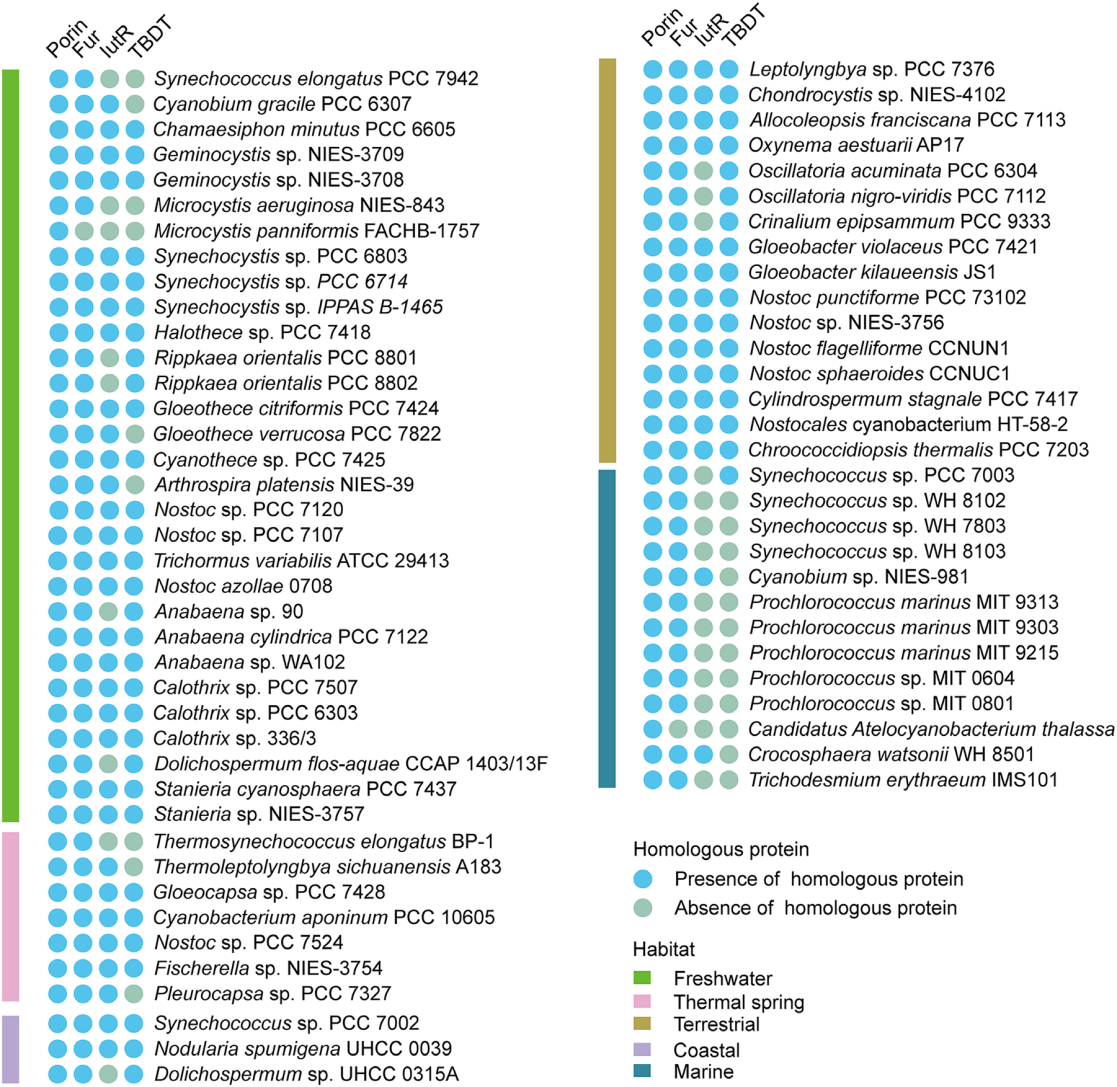
The distribution of the homologous proteins of porin, Fur, IutR, and TBDT in different cyanobacterial species from different habitats. Protein sequence alignment was conducted on the KEGG website, and an expectation value (*E* value) ≤ 1 × 10^−8^ was set to indicate the presence of homologous proteins.

Bioinformatic analysis indicated that IutR homologs are widely distributed and highly conserved in most freshwater and coastal cyanobacteria species, such as *Calothrix*, *Chroococcidiopsis*, *Leptolyngbya*, *Nostoc*, *Synechocystis*, *Microcystis*, and *Synechococus* but rarely exist in the representative open-ocean cyanobacteria with smaller genomes, such as *Prochlorococcus* and *Trichodesmium* (fig. S7 and table S3). The distribution characteristics of IutR are highly similar to energy-consuming transport pathways, such as TBDT (*19*), but different from FurA and porin (involved in passive transport), which are widely distributed in all cyanobacteria (Fig. 7). Considering the varying concentration of Fe in freshwater and coastal regions, compared to open oceans, it was speculated that the difference in the distributions of IutR or TBDT and FurA or porin may be attributed to the higher requirements of Fe homeostasis regulation in freshwater and coastal cyanobacteria. Consistently, IutR binding motifs were found in the promoter sequences of *tbdt* but not in that of porin-encoding genes (Table 3), which indicated that IutR might be involved in the regulation of the active transport pathway, not passive transport. This suggests that the IutR-mediated regulation of the active transport pathway is an important strategy for cyanobacterial adaption to complex and variable Fe environments.

## MATERIALS AND METHODS

### Cyanobacterial strains, culture conditions, and general methods

A motile, glucose-tolerant substrain of the model cyanobacterium *Synechocystis* sp. PCC 6803 originally obtained from J. Zhao’s laboratory (Peking University, China) was cultured in BG11 medium and shaken three times daily, at 30°C under continuous illumination (30 μmol photons m^−2^ s^−1^). BG11 plate medium was prepared by adding 0.3% Na_2_S_2_O_3_, 1.5% agar, and 8 mM *N*-tris(hydroxymethyl)methyl-2-aminoethanesulfonic acid (pH 8.2) (*72*). For the mutants and complementation strains, kanamycin (30 μg ml^−1^), spectinomycin (30 μg ml^−1^), gentamicin (5 μg ml^−1^), or erythromycin (15 μg ml^−1^) was added to BG11 medium.

The experimental materials used for the Fe-deplete assay, including the glassware, tips, and bottles, were soaked in 6 M HCl for at least 12 hours and rinsed with Milli-Q water six times to remove the residual Fe (*15*). All strains used for the Fe-deplete assay were grown to the logarithmic growth phase and harvested by centrifugation following two washes with Fe-deplete BG11 medium to remove extracellular Fe.

The growth of all cyanobacterial strains was assessed by measuring turbidity [OD_730_ (optical density at 730 nm)] from three independent biological replicates every 2 days until the 10th day. All samples were diluted threefold before measurement to ensure that the measured absorbance reading was below 1.5. Therefore, the OD_730_ values of all strains shown in the figures were threefolds of the absorbance we measured. To maintain the normal growth of cyanobacterial strains and avoid measurement errors caused by cell sedimentation, all the samples were shaken three times every day. Then, the ratios of the growth rates between the mutants and the wild-type strain were calculated. The statistics analysis method was used with an independent sample *t* test. To determine Chl a content, the absorption at 665 nm of methanol extracts was measured using an ultraviolet (UV) spectrophotometer and calculated according to the formula: Chl a (μg ml^−1^) = 12.95*A_665_ (*73*).The ETR and the maximum photochemical efficiency (F_v_/F_m_) of PSII were measured using a WATER-PAM chlorophyll fluorimeter (Walz, Germany). All samples were kept in the dark for 10 min before measurements (*74*). To determine the cyclic electron flow, 10 μM 3-(3,4-dichlorophenyl)-1,1-dimethylurea was added to the samples before measurements.

### Construction of the mutants and complementation strains of *Synechocystis* sp. PCC 6803

*Synechocystis* sp. PCC 6803 mutants were constructed using standard homologous recombination methods (*15*, *72*). For example, to create the *iutR1* mutant, the IutR1-encoding gene was amplified from the genomic DNA of *Synechocystis* sp. PCC 6803 and cloned into a pMD18-T vector (TaKaRa). Then, a kanamycin resistance cassette obtained from the plasmid pRL446 was inserted into the *Bal* I site of *iutR1* (fig. S4A). *Synechocystis* mutants were generated by natural transformation with corresponding plasmids as previously described (*22*). For the construction of the *iutR* triplex mutant, once a single mutant was obtained by homologous recombination, another recombinant plasmid was transformed into the mutant until we obtained the strain with all three genes successfully knocked out. For the construction of the *iutR* complementation mutants, the *iutR* gene was amplified from the genomic DNA of *Synechocystis* sp. PCC 6803 and cloned into PpsbAII expression vector by inserting into the *NdeI* site (*72*). The resulting plasmids were used to transform the gene fragments into the genome of *Synechocystis* 6803 mutant strains by homologous recombination methods. For the Com-*iutR1*–3× Flag strain, the 3× Flag tag was fused to the C-terminal of *iutR1*. PCR results confirmed that all mutants and complementation strains were completely segregated (fig. S4D). The primers used are listed in table S1, and constructed plasmids and strains are listed in table S2.

### Determination of cellular Fe content

The Fe content of the cells was measured according to the methods described previously (*15*). Briefly, exponential cells were cultured for 4 days, collected by centrifugation, washed twice with 10 mM EDTA, and then washed once with Milli-Q water to remove extracellular Fe. Then, the samples were digested with 80% HNO_3_ in a microwave oven and reconstituted in a fresh 5% HNO_3_ solution. The Fe content of the cells was determined using an atomic absorption spectrometer (AA240FS, Varian).

### RNA extraction and RT-qPCR

The *Synechocystis* sp. PCC 6803 strains were grown in standard BG11 medium for 3 days, and then cultured in Fe-replete or Fe-deplete BG11 medium for 24 hours. About 50 ml of suspension was collected by centrifugation and quickly frozen in liquid nitrogen. The total RNA was extracted using a FastPure Universal Plant Total RNA Isolation Kit (Vazyme Biotech Co. LTD, China) following the manufacturer’s instructions. PrimeScript RT Reagent Kit with gDNA Eraser (TaKaRa, Japan) was used to eliminate genomic DNA and RNA reverse transcription. The RT-qPCR assay was performed with a 7900HT Fast real-time PCR system (Thermo Fisher Scientific, USA) using SYBR Green Real-Time PCR Mix following the manufacturer’s instructions (Cowin Biotechnology Co. LTD, China). All the primers used for RT-qPCR are listed in table S1. Relative quantification of transcripts was estimated using the 2^-∆∆CT^ method, and the transcript abundance of each gene was calculated relative to the expression of a reference gene *rnpB*.

### Yeast two-hybrid assay and yeast one-hybrid assay

In the yeast two-hybrid assay, the protein-protein interactions between IutR proteins were detected using the Matchmaker GAL4 Two-Hybrid System 3 (Clontech, Palo Alto, CA, USA). The gene fragments of interest were inserted into pGBKT7 and pGADT7, respectively. The resultant plasmids were cotransformed into *Saccharomyces cerevisiae* AH109 and cultured on SD/-Trp-Leu-His agar plates for selection. The selected positive transformants were then transferred onto SD/-Trp-Leu-His-Ade plates and incubated at 28°C for 3 days.

In the yeast one-hybrid assay, the predicted promoter region of three *tbdt* was recombined into the *Bst*I-digested yeast reporter vector pAbAi, and the resulting plasmid was transformed into the Y1HGold yeast bait strain. The construction of pGADT7-*iutR* is the same as the yeast two-hybrid assay. The pGADT7 empty vector was used as a negative control. The growth of yeast cells on the SD plates minus leucine with 300 ng ml^−1^ of aureobasidin A (AbA; Solarbio, China) indicates the interaction between IutR and *tbdt* promoter.

### Electrophoretic mobility shift assays

The EMSA assay that verifies the interactions between IutR and the promotors of potential target genes was performed as described previously. DNA fragments composed of biotin-labeled DNA, nonbiotin-labeled DNA, and nonspecific competitors were prepared by PCR with the primers listed in table S1. Recombinant glutathione *S*-transferase (GST)–tagged IutR from *E. coli* cells were purified. The binding reactions were performed in the EMSA-binding buffer [10 mM tris-HCl (pH 7.5), 50 mM KCl, 1 mM dithiothreitol, 0.1% bovine serum albumin (BSA), and 5% w/v glycerol] for 30 min at 30°C. Two hundred nanograms of GST protein was added as the negative control, and 200 ng of unlabeled DNA and 2000 ng of poly (deoxyinosinic-deoxycytidylic) acid were added as specific and unspecific competitors, respectively. The reaction mixtures were separated on 6% polyacrylamide gels at 80 V for 2 hours in the 1× tris-borate EDTA (TBE) buffer at 4°C. After electrophoresis, the gels were soaked with 0.5× TBE buffer and then transferred to nylon membranes (Millipore) at 20 V for 1 hour using Trans-Blot SD (Bio-Rad). After UV cross-linking for 10 min, the nylon membranes were soaked with 5% dried skimmed milk for more than 12 hours and incubated with HRP-conjugated streptavidin for 1 hour. Then, the samples were visualized with ECL solution (Yeasen, China) using FluorChem M (ProteinSimple).

The interactions between IutR and promotors of *furA* and *perR* were assessed using a modified EMSA assay procedure. DNA fragments were prepared by PCR with the primers listed in table S1. The binding reactions were incubated in the EMSA-binding buffer [10 mM tris-HCl (pH 7.5), 50 mM KCl, 5 mM MgCl_2_, and 10% w/v glycerol] for 30 min at 30°C. Two hundred nanograms of BSA protein was added as the negative control. The reaction mixtures were separated on 6% polyacrylamide gels at 90 V for 1 hour in the 1× TB buffer at 4°C. After electrophoresis, the gels were soaked with 20,000× GelRed (Accurate Biotechnology Co. LTD, China) and then were visualized using the Tanon 3500 Gel Imaging System (Tanon, Shanghai, China).

### Sample preparation, RNA extraction, and transcriptome sequencing

The *Synechocystis* sp. PCC 6803 strains were grown in standard BG11 medium for 3 days, and then cultured in Fe-deplete (10 μM FeCl_3_) or Fe-deplete (10 μM FeCl_3_ and 20 μM desferrioxamine B) BG11 medium for 24 hours at 30°C under continuous illumination (30 μmol photons m^−2^ s^−1^). About 50 ml of suspension was collected by centrifugation (5 min, 5000 rpm, 4°C) and quickly frozen in liquid nitrogen. RNA was extracted from the sample using a TRIzol Reagent Kit (Invitrogen, CA, USA) according to the manufacturer’s instructions. Residual DNA was removed using the PrimeScript RT Reagent Kit with gDNA Eraser (TaKaRa, Japan). Libraries were prepared and sequenced by the gene created using the low-input protocol for total RNA. In brief, reverse transcription and library preparation included the following steps: fragmentation of RNA using ribonuclease III, reverse transcription of RNA fragments, and ligation of the resulting cDNA fragments to adaptors. Raw data were obtained by Illumina NovaSeq 6000. The impurities in raw data were removed to obtain clean data, followed by reads expression statistics, sequencing saturation analysis, gene annotation, and screening of DEGs. For all detected gene transcript level statistics, the number of significantly different expression genes under Fe-deplete conditions was counted in the presence of a 1.5- or 0.67-fold difference, *P* < 0.1.

### Hi-C library construction and analysis

To construct the Hi-C library, *Synechocystis* sp. PCC 6803 strains were grown in Fe-replete BG11 medium for 3 days, and then cultured under Fe-replete and Fe-deplete conditions for 24 hours. Then, the cells were harvested and cross-linked with 3% formaldehyde for 1 hour at 4°C and quenched with 375 mM glycine for 15 min. The cross-linked cells were lysed by lysozyme at 37°C for 10 min. After treatment with 0.5% SDS to inactivate the endogenous nucleases, chromatin DNAs were digested with 50 U Sau3AI, marked with biotin-14-dCTP, and then ligated by using 50 U T4 DNA ligase. After reversing the cross-links, the ligated DNA was extracted using the QIAamp DNA Mini Kit. The purified DNA was cleaved into fragments of 300 to 500 bp. Subsequently, the DNA fragments were blunt-end repaired, A-tailed, and combined with an adaptor. Next, the DNA fragments were pulled down through biotin-streptavidin and amplified by PCR. Last, the Hi-C libraries were quantified and sequenced on the HiSeq X Ten platform.

The Hi-C library data were analyzed by using the procedures described previously (*48*). Briefly, the Hi-C sequencing data were quality-filtered and mapped to the genome of *Synechocystis* sp. PCC 6803 using the ICE software (*75*). The valid pairs were binned into 10-kb nonoverlapping genomic intervals to generate contact maps. These contact maps were normalized by using an iterative normalization method that eliminated the systematic biases. The locations of CIDs were determined at 2-kb resolution using the insulation score method (*76*). Fit-Hi-C software (*77*) was used to calculate the significance of interactions at 1-kb resolution. The interactions with *P* ≤ 0.01, *Q* ≤ 0.01, and the contact count >2 were identified as significant interactions. Chromosomal 3D structures were constructed at 10-kb resolution using the PASTIS software (*78*).

### ChIP-seq assay

To perform ChIP-seq assays, a recombinant plasmid that expresses IutR1 fused with a 3× Flag tag was constructed, and then transformed into the *iutR* triplex mutant strain (Mut 3*iutR*). The resulting Com-*iutR1*–3× Flag cyanobacterial strain was cultured in Fe-replete BG11 medium for 3 days and then subjected to Fe deficiency (0 nM Fe) for 24 hours. For the ChIP-seq assay, cells were rinsed with chilled PBS and frozen quickly using liquid nitrogen. Then DNA was extracted for library construction and sequenced using Illumina NovaSeq 6000 (SEQHEALTH, Wuhan, China). The clean data were aligned to the reference genome of *Synechocystis* sp. PCC 6803, and the peaks were identified using MACS2. Hypergeometric Optimization of Motif EnRichment (HOMER; http://homer.ucsd.edu/homer/motif/) was used to analyze the binding motifs, using bound sequences as the background sequence.

### Statistical analysis

Statistical analysis was performed using IBM SPSS Statistics software. For all statistics in this study, the error bar is STDEV. Analyses were considered statistically significant if adjusted *P* < 0.05.

## Supplementary Materials

### This PDF file includes:

Figs. S1 to S8 Tables S1 to S3 Legends for movies S1 and S2 References
