# Supplementary material for: A global transcriptional activator involved in the iron homeostasis in cyanobacteria
Source: Sci Adv. 2024 Jul 3;10(27):eadl6428. doi: 10.1126/sciadv.adl6428 (PMC11221513; doi:10.1126/sciadv.adl6428)
Supplement: Supplementary file 1 — Figs. S1 to S8 Tables S1 to S3 Legends for movies S1 and S2 References [file sciadv.adl6428_sm.pdf]

Science Advances
AAAS

Supplementary Materials for

A global transcriptional activator involved in the iron homeostasis in cyanobacteria

Ling-Mei Liu *et al.*

Corresponding author: Weizhong Chen, chenweizhong@nbu.edu.cn; Hai-Bo Jiang, jianghaibo@nbu.edu.cn

Sci. Adv. **10**, eadl6428 (2024)
DOI: 10.1126/sciadv.adl6428

The PDF file includes:

Figs. S1 to S8
Tables S1 to S3
Legends for movies S1 and S2
References

Other Supplementary Material for this manuscript includes the following:

Movies S1 and S2

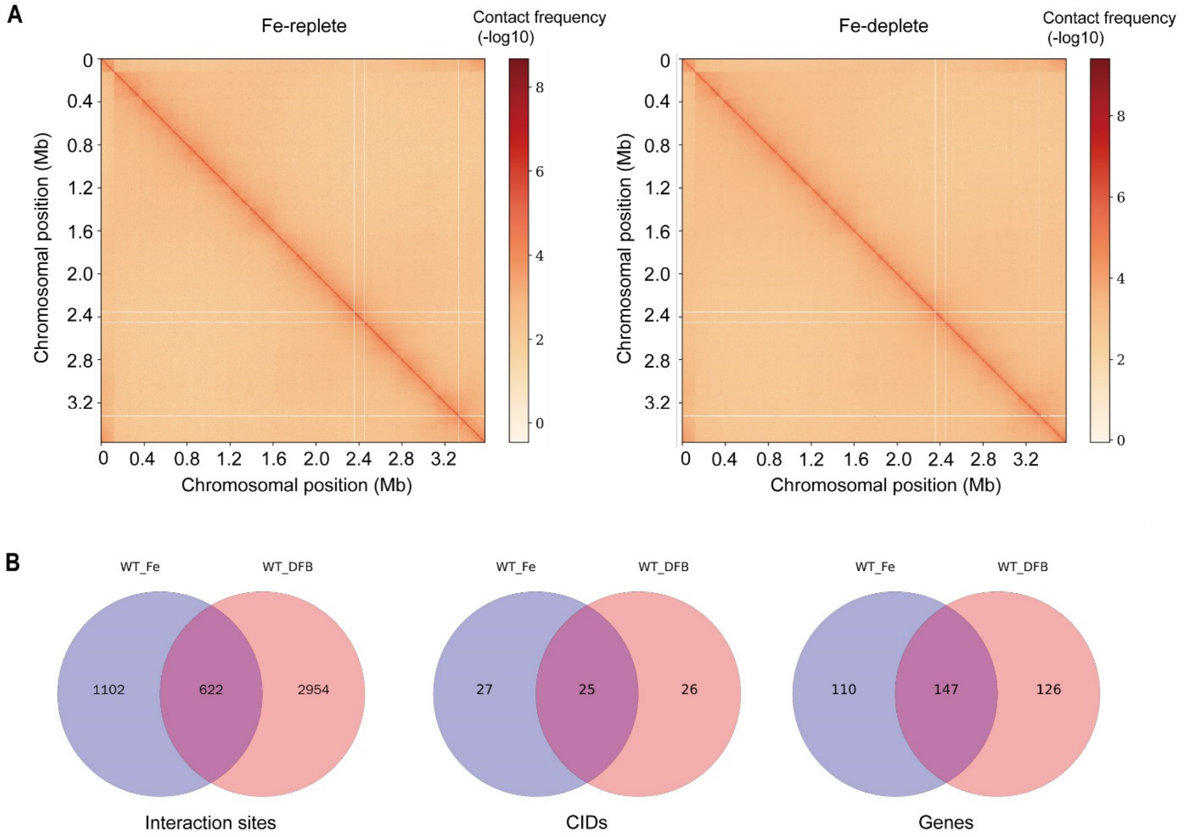

Figure S1. The Hi-C results of *Synechocystis* sp. PCC 6803 under Fe-replete and Fe-deplete conditions. (**A**) Normalized Hi-C contact map for *Synechocystis* sp. PCC 6803 displaying contact frequencies for pairs of 10-kb bins across the genome under Fe-replete and Fe-deplete conditions. (**B**) Identification of interaction sites, CIDs, and genes in CID boundary under Fe-replete (WT_Fe) and Fe-deplete (WT_DFB) conditions.

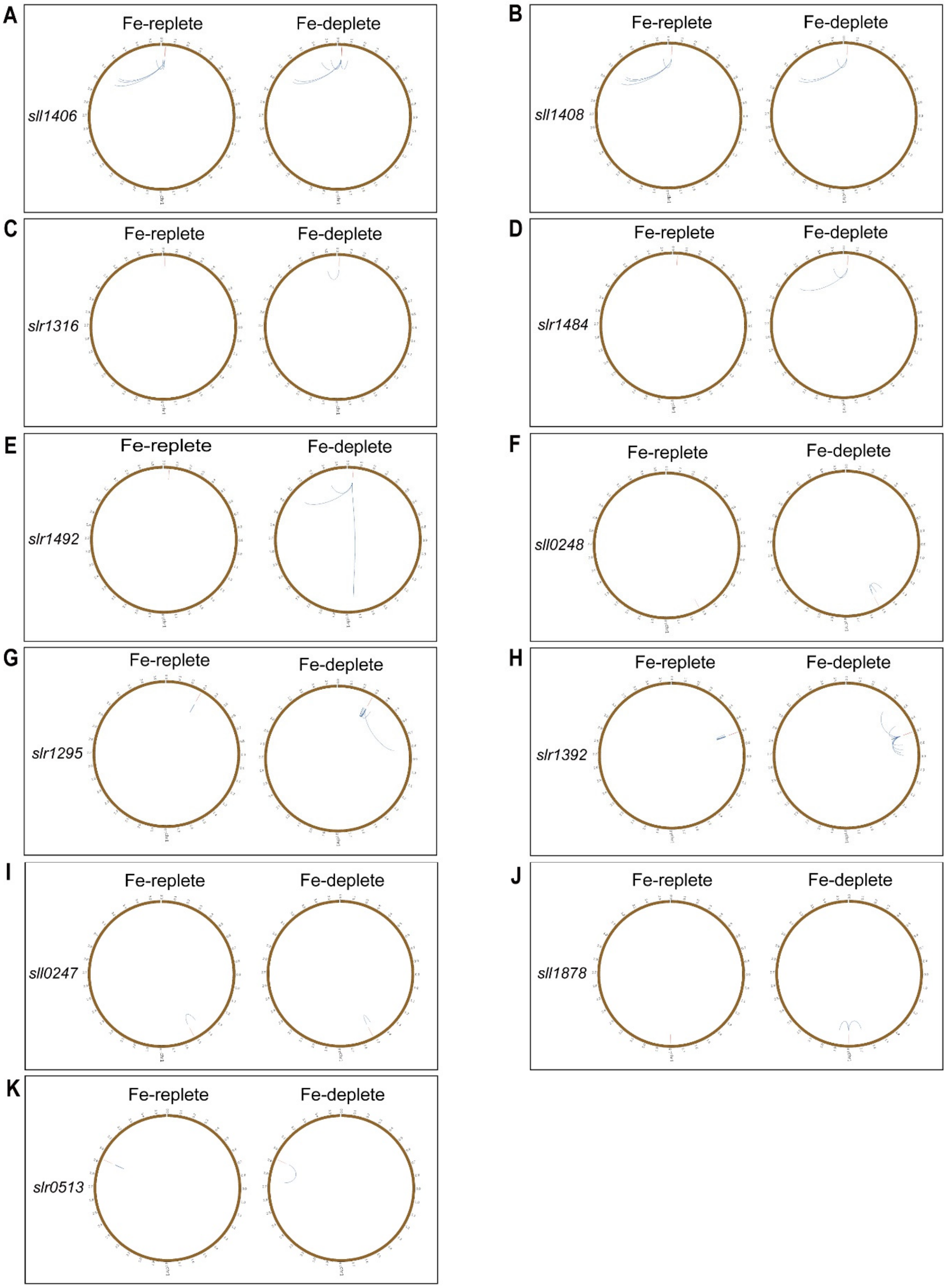

Figure S2. The single genes involved in the low Fe adaption with significant enhanced interactions under Fe-deplete conditions comparing with Fe-replete conditions. (A) *sll1406* gene, **(B)** *sll1408* gene, **(C)** *slr1316* gene, **(D)** *slr1484* gene, **(E)** *slr1492* gene, **(F)** *sll0248* gene, **(G)** *slr1295* gene, **(H)** *slr1392* gene, **(I)** *sll0247* gene, **(J)** *sll1878* gene, **(K)** *slr0513* gene.

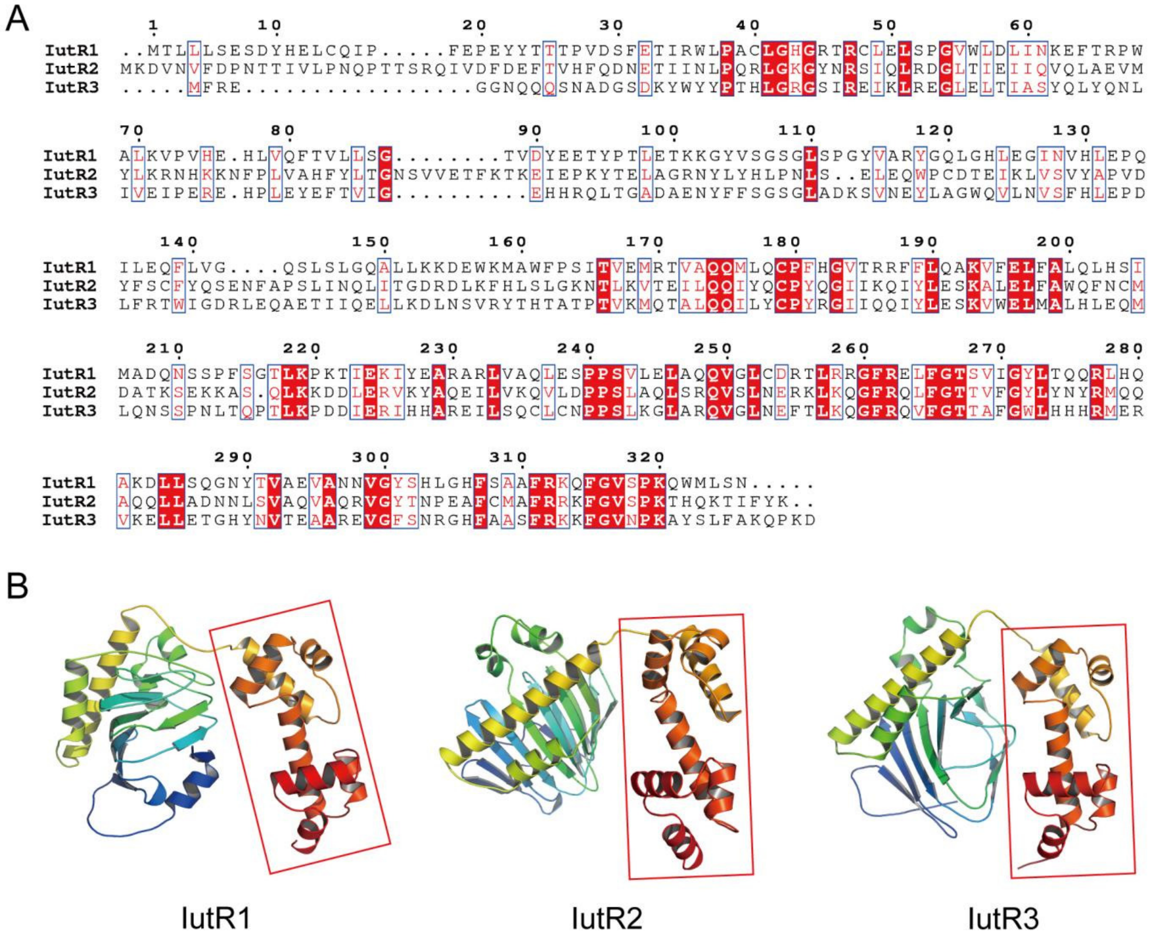

Figure S3. The sequence and structure of three IutR proteins in *Synechocystis* sp. PCC 6803. (**A**) The sequence alignment of three IutR proteins. The multiple sequence alignment was performed by using Clustal Omega, and visualized in ESPript 3. (**B**) The structure of three IutR proteins predicted by alphafold2. The typical transcription factor domain Helix-Turn-Helix (HTH) was labeled with red box.

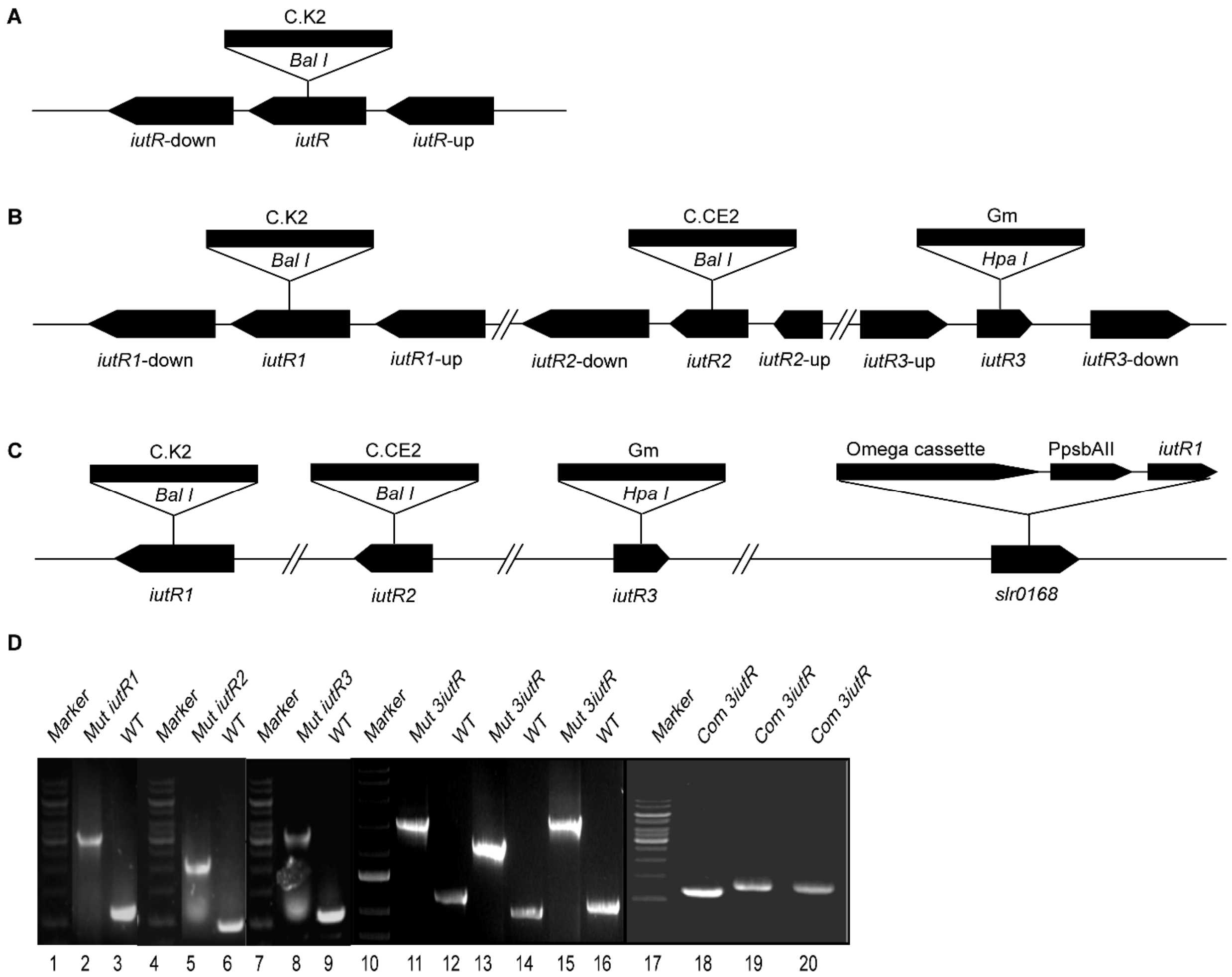

Figure S4. Construction of *iutR* single mutant, *iutR* triplex mutant and *iutR* complementation strains. (**A**) Sketch map for the construction of *iutR* single mutants. (**B**) Sketch map for the construction of *iutR* triplex mutant. (**C**) Sketch map for the construction of *iutR* complementation strains. (**D**) PCR verification of *iutR* single mutants, *iutR* triplex mutant and *iutR* complementation strains.

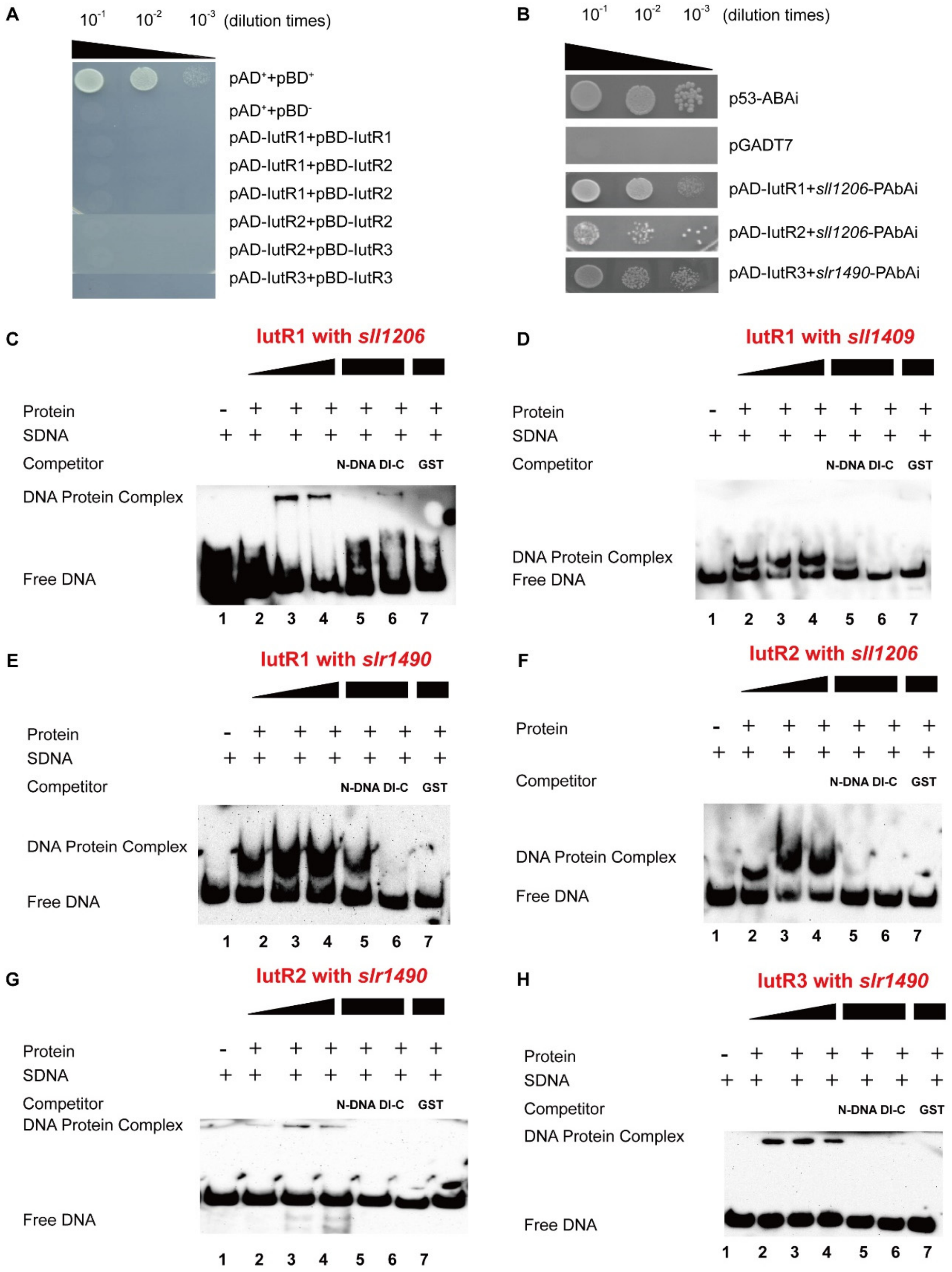

Figure S5. Assessment of the protein-protein and protein-DNA interactions of IutR proteins. (**A**) Yeast two-hybrid assay was performed to confirm the interaction between three different IutRs. The yeast transformants containing positive control plasmids (pAD+, pBD+), negative control plasmids (pAD+, pBD-) and plasmids for testing protein interaction were grown on SD/-Trp-Leu-His-Ade plate for 3 days. 10 µl of cell suspension was diluted from OD_{600} 0.1 to 0.001, indicated as the above triangles from left to right. (**B**) Yeast one-hybrid assay was performed to confirm the interactions between IutRs and *tbdt* promoters. The yeast transformants containing positive control plasmids (p53-ABAi), negative control plasmids (pGADT7^{-}) and plasmids for testing protein-DNA interaction were grown on urea plates containing aureobasidin A. (**C-H**) Electrophoretic mobility shift assay (EMSA) of IutRs interacting with different *tbdt* promoters. (**C**) IutR1 with *sll1206*, (**D**) IutR1 with *sll1409*, (**E**) IutR1 with *slr1490*, (**F**) IutR2 with *sll1206*, (**G**) IutR2 with *slr1490*, (**H**) IutR3 with *slr1490*.

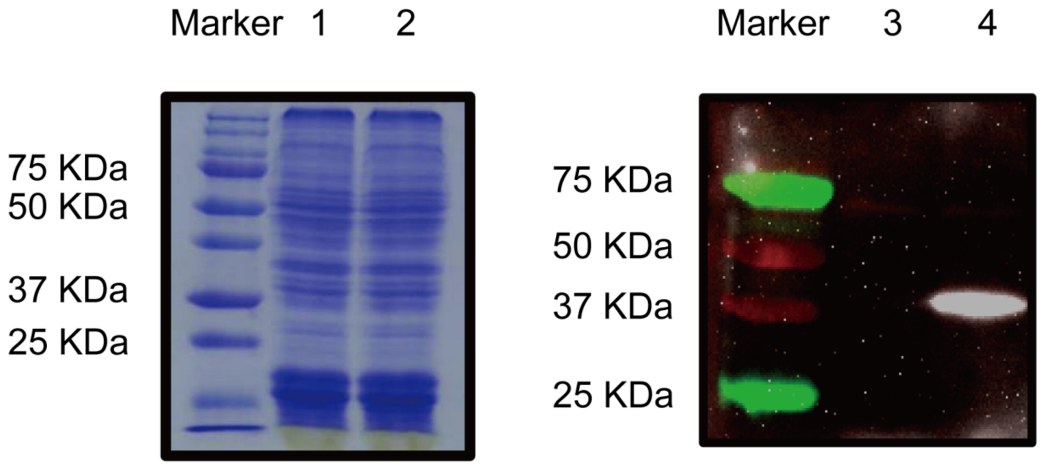

Figure S6. SDS-PAGE (left panel) and western blotting (right panel) results of wild-type strain and Com-*iutR1*-3×Flag strains. Lane 1 and 3, wild-type strain; Lane 2 and 4, Com-*iutR*-3Flag strains.

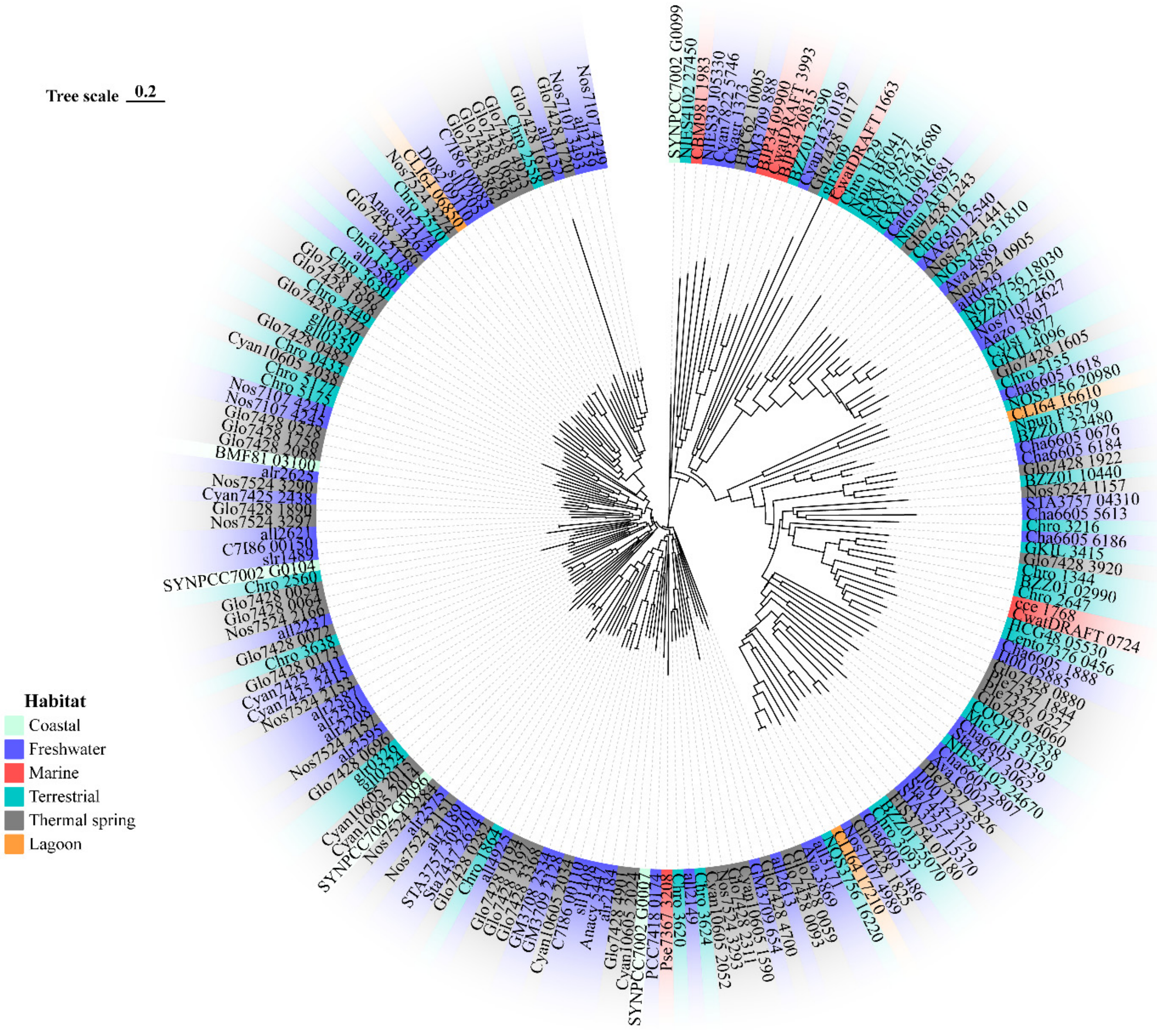

Figure S7. Phylogenetic analysis of Sll1205 in selected cyanobacterial species by a *Synechocystis* sp. PCC 6803 homolog. Representative species were selected and a maximum likelihood phylogenetic tree was generated by RAxML v8.1.20 using a protein sequence alignment generated at the MAFFT website.

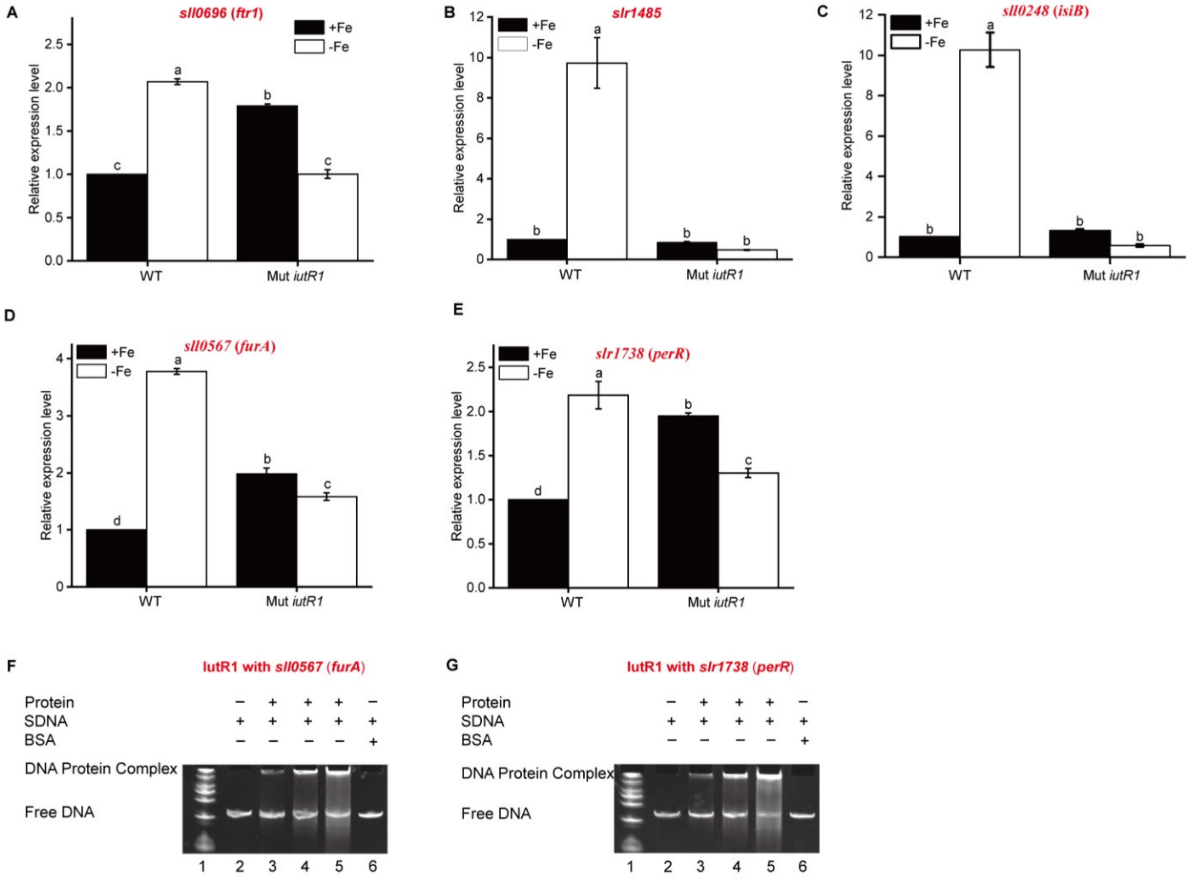

Figure S8. Transcription analysis of Fe uptake-related genes in *Synechocystis* sp. PCC 6803 by RT-qPCR and assessment of the protein-DNA interactions of IutR1. (**A-E**) Transcription levels of Fe-uptake related genes in the wild-type strain (WT) and *iutR1* single mutant cultured in Fe-replete (+Fe) and Fe-deplete (-Fe) BG11 medium for 24 h. Five genes, including *sll0696* (**A**), *slr1485* (**B**), *sll0248* (**C**), *sll0567* (**D**) and *slr1738* (**E**) were measured. The final Fe concentrations were 21.4 µM and 0 nM for the Fe-replete and Fe-deplete conditions, respectively. (**F and G**) Electrophoretic mobility shift assay (EMSA) of IutR1 interacting with promoters of *furA* (**F**) and *perR* (**G**).

Table S1 Primers used in this study

Primers	Sequences	Usages
sll1205-1	5’-GATAATCCACAAGGTTGTCCCAATCGC-3’	a
sll1205-2	5’-CAGCATCCACTGTTTAGGGCTGACACC-3’	a
sll1408-1	5’-GATGTTAATGTATTTGATCCCAACACCACG-3’	a
sll1408-2	5’-CTGGATTGGTATATCCTACCCTTTGTGCT-3’	a
slr1489-1	5’-GTGATGGCTTGACGCATCGGCTTCAC-3’	a
slr1489-2	5’-GAGGCGGCAAAATGTCCCCGATTG-3’	a
sll1205-com-1	5’-ATGACCCTACTTCTTTCCGAGTCCGAC -3’	b
sll1205-com-2	5’-TCAATTACTCAGCATCCACTGTTTAGGGC-3’	b
sll1408-com-1	5’-ATGAAAGATGTTAATGTATTTGATCCCAACACCAC -3’	b
sll1408-com-2	5’-CTATTTGTAGAAAATTGTCTTTTGATGGGTTTTGGG-3’	b
slr1489-com-1	5’-ATGTCGATCTTTTTTTCTCAATCTGATTACTTGGC-3’	b
slr1489-com-2	5’-TTAGTCTTTAGGTTGTTTGGCAAACAATGAATAGG-3’	b
sll1205-3F-1	5’-<br>ATGACCCTACTTCTTTCCGAGTCCGACTACCATGAGCTTTGTCAAA<br>TT-3’	c
sll1205-3F-2	5’-<br>TTAACCGGGAGAGACTATCACTTGTCGTCGTCGTCCTTGTAGTCCT<br>TGTCGTCGTCGTCCTTGTAGTCCTTGTCGTCGTCGTCCTTGTAGTCA<br>TTACTCAGCATCCACTGTT-3’	c
sll1206-P-1	5’-GAAGTCGACTTGAGAAGATCTACGAAGCGAG-3’	d
sll1206-P-2	5’-GCAGGTACCTTTAACCGGGAGAGACTATCTATC-3’	d
sll1409-P-1	5’-TATGTCGACCTCGAGCAATGGCCATG-3’	d
sll1409-P-2	5’-GCGAGCGGTACCTTACTGATAATTAATGGAT-3’	d
slr1490-P-1	5’-GCGTCGACGGGAATTATCCAACAGATTTATC-3’	d
slr1490-P-2	5’-CCGCGGTACCCAAAGCTTGAAATTCTTA-3’	d
sll1205-AD-1	5’-CGTCATATGACCCTACTTCTTTCCGAGTCC-3’	e
sll1205-AD-2	5’-CAGCTCGAGTCAATTACTCAGCATCCACTGT-3’	e
sll1408-AD-1	5’-GGCCGCATATGAAAGATGTTAATGTATTTGATCC-3’	e
sll1408-AD-2	5’-GCCGGAATTCCTATTTGTAGAAAATTGTCTTTTG-3’	e
slr1489-AD-1	5’-GCGGCATATGTCGATCTTTTTTTCTCAATCTG-3’	e
slr1489-AD-2	5’-GGTCTCGAGTTAGTCTTTAGGTTGTTTGGC-3’	e
sll1205-BD-1	5’-GTCCATATGATGACCCTACTTCTTTCCGAG-3’	e
sll1205-BD-2	5’-AAGCTGCAGTCAATTACTCAGCATCCACTG-3’	e
sll1408-BD-1	5’-TGTTTCCGCTGCAGCTATTTAGAAAATTGTC-3’	e
sll1408-BD-2	5’-CCGCTGCAGCTATTTGTAGAAAATTGTCTTT-3’	e
slr1489-BD-1	5’-CGGCATATGATGTCGATCTTTTTTTCTCAATCT-3’	e
slr1489-BD-2	5’-AAGCTGCAGTTAGTCTTTAGGTTGTTTGGC-3’	e
41a-*sll1205*-1	5’-TAAGCTAGCATGACCCTACTTCTTTCCGAG-3’	f
41a-*sll1205*-2	5’-GCACTCGAGTCAATTACTCAGCATCCACT-3’	f

41a-*sll1408*-1	5’-TGCGCTAGCATGAAAGATGTTAATGTATTTGATCC-3’	f
41a-*sll1408*-2	5’-GCCGTCGACCTATTTGTAGAAAATTGTCTTTTG-3’	f
41a-*slr1489*-1	5’-GGCGCTAGCATGTCGATCTTTTTTTCTCAAT-3’	f
41a-*slr1489*-2	5’-GCACTCGAGTTAGTCTTTAGGTTGTTTGGC-3’	f
B-E-*slr1206*-1	5’-biotin-labeled GAAGTCGACTTGAGAAGATCTACGAAGCGA-3’	f
B-E-*sll1409*-1	5’-biotin-labeled TATGTCGACCTCGAGCAATGGCCATG-3’	f
B-E-*slr1490*-1	5’-biotin-labeled GCGTCGACGGGAATTATCCAACAGATTTATC-3’	f
B-E-*isia*-1	5’-biotin-labeled TCAGTCGACAGATTTACCTACGTTAGGGA-3’	f
N-E-*slr1206*-1	5’-GAAGTCGACTTGAGAAGATCTACGAAGCGA-3’	f
N-E-*sll1409*-1	5’-TATGTCGACCTCGAGCAATGGCCATG-3’	f
N-E-*slr1490*-1	5’-GCGTCGACGGGAATTATCCAACAGATTTATC-3’	f
N-E-*isia*-1	5’-TCAGTCGACAGATTTACCTACGTTAGGGA-3’	f
E-*slr1206*-2	5’-GCAGGTACCTTTAACCGGGAGAGACTATC-3’	f
E-*sll1409*-2	5’-GCGAGCGGTACCTTACTGATAATTAATGGAT-3’	f
E-*slr1490*-2	5’-CCGCGGTACCCAAAGCTTGAAATTCTTA-3’	f
E-*isia*-2	5’-GGCGGTACCAGAATTGCCTCCTTAATT-3’	f
E-*sll0567*-1	5’-TGGCTGCATCCACCACCAATGG-3’	f
E-*sll0567*-2	5’-GTTCTATGGGGGCGCTTAGAAGCAG-3’	f
E-*slr1738*-1	5’-GGTCATGGTTACCTCTGGCTATCTAAACG-3’	f
E-*slr1738*-2	5’-ACTGTTCCTCCTAACATCGGTGCG-3’	f
RT-*sll1205*-1	5’-TGAACCTGAGTATTACACCACCACTCCAG-3’	g
RT-*sll1205*-2	5’-GGCCACTCCCGGATACATAACCCTT-3’	g
RT-*sll1408*-1	5’-CGATCTATCCAGTTGAGGGACGGCTT-3’	g
RT-*sll1408*-2	5’-CAGTATCACATGGCCATTGCTCGAGTTC-3’	g
RT-*slr1489*-1	5’-ACTTGGCCATGTTCAGGGAAGGAGG-3’	g
RT-*slr1489*-2	5’-TCTGCATCAGCCCCGGTTAACTGAC-3’	g
RT-*sll1406*-1	5’-CAACATAGTCCGCATTACGGTGACGG-3’	g
RT-*sll1406*-2	5’-GCACTGGGCACAAAGTAGCTAGCTTCC-3’	g
RT-*sll1206*-1	5’-CGATCGCCTCTCCGAACCTACAAACG-3’	g
RT-*sll1206*-2	5’-GGTCGAATCAGCAATTGTAACCCTTGCG-3’	g
RT-*sll1409*-1	5’-ACGGCCGATCAATCCCAGCTAATACC-3’	g
RT-*sll1409*-2	5’-GGAACAGTACCATTGCCAGTTACGGTAATG-3’	g
RT-*slr1490*-1	5’-GACCGAAGCTGGACTGGAACTGACC-3’	g
RT-*slr1490*-2	5’-GGGATTGGTCTGTGGCATTAACTTCAACC-3’	g
RT-*slr1295*-1	5’-ATGGTCCAAAAGTTATCCCGTCGCC-3’	g
RT-*slr1295*-2	5’-TGTTGGCCCCTTCAGACTTAATCCGT-3’	g
RT-*slr0513*-1	5’-ATGACAACTAAGATTTCCCGGCGGAC-3’	g
RT-*slr0513*-2	5’-CCGCCATAACATACCGGCATCTACGG-3’	g
RT-*slr0964*-1	5’-TTTTCATTGTCACCCTGCGG-3’	g
RT-*slr0964*-2	5’-CCCAGCCTTTGAGTTGCATT-3’	g
RT-*slr1485*-1	5’-TTCCGGCACTGTTGTTCAAG-3’	g

RT-*slr1485*-2	5'-TTCATACTCTCCTCCAGCGG-3'	g
RT-*sll0248*-1	5'-CTCAAACCGGCAACACTGAA-3'	g
RT-*sll0248*-2	5'-TCATCAACATCAGCCTGGGA-3'	g
RT-*sll0567*-1	5'-CGAACTTAATGCCCGTGGTT-3'	g
RT-*sll0567*-2	5'-GTGTAACTCCTCGGCACTGA-3'	g
RT-*slr1738*-1	5'-CACGGTGTACAGTTCCCTCA-3'	g
RT-*slr1738*-2	5'-ATCCACATCCTCTATCGCCC-3'	g

a, used for construction of mutants; b, used for construction of complementation strains； c, used for construction of complementation strains with 3Flag; d, used for construction of plasmid for yeast one-hybrid assay; e, used for construction of plasmid for yeast two-hybrid assay; f, used for EMSA assay; g, used for RT-qPCR assay.

Table S2 Cyanobacterial strains and plasmids used in this study

Strains and Plasmid	Derivation and/or relevant characteristics[a]	Reference or Source
***Synechocystis* strains**		
Synechocystis sp. PCC 6803	Wild type	This study
Mut-*iutR1*	Km^r, *Synechocystis* 6803 mutant, result of transformation with HS-S-P-1	This study
Mut-*iutR2*	Km^r, *Synechocystis* 6803 mutant, result of transformation with HS-S-P-2	This study
Mut-*iutR3*	Km^r, *Synechocystis* 6803 mutant, result of transformation with HS-S-P-2	This study
Mut-3*iutR*	Km^r, Em and Gm, *Synechocystis* 6803 mutant, result of transformation with HS-S-P-1, HS-S-P-2 and HS-S-P-3 one by one	This study
Com-*iutR1*	Km^r, *Synechocystis* 6803 mutant, result of transformation with HS-S-P-4	This study
Com-*iutR2*	Km^r, *Synechocystis* 6803 mutant, result of transformation with HS-S-P-5	This study
Com-*iutR3*	Km^r, *Synechocystis* 6803 mutant, result of transformation with HS-S-P-6	This study
Com-*iutR1*-3×Flag	Km^r, *Synechocystis* 6803 mutant, result of transformation with HS-S-P-7	This study
**Plasmids**		
HS-S-P-1	Amp^rKm^r, PCR fragment containing the *sll1205* gene cloned into pMD18-T, and C.K2 inserted in its *Bal* I site	This study
HS-S-P-2	Amp^rKm^r, PCR fragment containing the *sll1408* gene cloned into pMD18-T, and C.CE2 inserted in its *Bal* I site	This study
HS-S-P-3	Amp^rKm^r, PCR fragment containing the *slr1489* gene cloned into pMD18-T, and Gm inserted in its *Hpa* I site	This study
HS-S-P-4	Amp^rSp^r, PCR fragment containing the *sll1205* gene cloned into *Omega-PpsbAII-0168*	This study
HS-S-P-5	Amp^rSp^r, PCR fragment containing the *sll408* gene cloned into *Omega-PpsbAII-0168*	This study
HS-S-P-6	Amp^rSp^r, PCR fragment containing the *slr489* gene cloned into *Omega-PpsbAII-0168*	This study
HS-S-P-7	Amp^rSp^r, PCR fragment containing the *sll1205* gene and 3Flags cloned into *Omega-PpsbAII-0168*	This study
HS-S-P-8	Amp^r, PCR fragment containing the *sll1205* gene cloned into pGADT7	This study
HS-S-P-9	Amp^r, PCR fragment containing the *sll1408* gene cloned into pGADT7	This study
HS-S-P-10	Amp^r, PCR fragment containing the *slr1489* gene cloned into pGADT7	This study
HS-S-P-11	Km^r, PCR fragment containing the *sll1205* gene cloned into pGBDT7	This study
HS-S-P-12	Km^r, PCR fragment containing the *sll1408* gene cloned into pGBDT7	This study
HS-S-P-13	Km^r, PCR fragment containing the *slr1489* gene cloned into pGBDT7	This study
HS-S-P-14	Amp^r, PCR fragment containing promter of *sl1206* cloned into PAbAi	This study

Strains and Plasmid	Derivation and/or relevant characteristics[a]	Reference or Source
HS-S-P-15	Amp^r, PCR fragment containing promter of *sl1409* cloned into PAbAi	This study
HS-S-P-15	Amp^r, PCR fragment containing promter of *slr1490* cloned into PAbAi	This study
HS-S-P-16	Km^r, PCR fragment containing the *sll1205* gene cloned into pET41a	This study
HS-S-P-17	Km^r, PCR fragment containing the *sll1408* gene cloned into pET41a	This study
HS-S-P-18	Km^r, PCR fragment containing the *slr1489* gene cloned into pET41a	This study
PRL446	Spr, cloning vector with a kanamycin resistance cassette omega	(79)
pHS298	Sp^r, PpsbAII-*slr0168*	This study
pRL57	Spr, cloning vector with a spectinomycin resistance cassette omega	(*80*)
pRL598	Cm^r Em^r, cloning vector with an erythromycin resistance marker (C.CE2)	(*79*)
pGADT7	Ap^r, yeast two-hybrid expression vector with ADH1 promoter and a fusionof GAL4 AD	Clontech
pGBKT7	Km^r, yeast two-hybrid expression vector a fusion of GAL4 DNA bindingDomain (DNA-BD)	Clontech
pGADT7-T	Apr, the T-antigen gene cloned into pGADT7	Clontech
pGBKT7-53	Kmr, the p53 gene cloned into pGBKT7	Clontech
pGBKT7-Lam	Kmr, the lamin C gene cloned into pGBKT7	Clontech

[a]Amp, ampicillin; Km, kanamycin; Sp, spectinomycin; Cm, chloramphenicol; Gm, gentamicin; Em, erythromycin.

Table S3 The number of putative IutR homologues in selected cyanobacterial species/strains.

Genera/Order	Cyanobacterial strain	Habitat	Number of IutR
Synechococcus	*Synechococcus* sp. PCC 7002	Coastal	4 results
	Synechococcus sp. PCC 7003	Marine	0 result
	Synechococcus elongatus PCC 7942	Freshwater	0 result
	Synechococcus sp. WH 8102	Marine	0 result
	Synechococcus sp. WH 7803	Marine	0 result
	Synechococcus sp. WH 8103	Marine	0 result
Thermosynechococcus	*Thermosynechococcus elongatus BP-1*	Thermal spring	0 result
Cyanobium	*Cyanobium gracile* PCC 6307	Freshwater	1 result
	Cyanobium sp. NIES-981	Marine	1 result
Dactylococcopsis	*Dactylococcopsis salina* PCC 8305	Freshwater	0 result
Chamaesiphon	*Chamaesiphon minutus* PCC 6605	Freshwater	9 results
Leptolyngbya	*Leptolyngbya* sp. PCC 7376	Terrestrial	1 result
Pseudanabaena	*Pseudanabaena* sp. PCC 7367	Marine	1 result
Prochlorococcus	*Prochlorococcus marinus* MIT 9313	Marine	0 result
	Prochlorococcus marinus MIT 9303	Marine	0 result
	Prochlorococcus marinus MIT 9215	Marine	0 result
	Prochlorococcus sp. MIT 0604	Marine	0 result
	Prochlorococcus sp. MIT 0801	Marine	0 result
Thermoleptolyngbya	*Thermoleptolyngbya sichuanensis* A183	Thermal spring	1 result
Gloeocapsa	*Gloeocapsa* sp. PCC 7428	Thermal spring	37 results
Geminocystis	*Geminocystis* sp. NIES-3709	Freshwater	3 results
	Geminocystis sp. NIES-3708	Freshwater	1 result
Chondrocystis	*Chondrocystis* sp. NIES-4102	Terrestrial	2 results
Microcystis	*Microcystis aeruginosa* NIES-843	Freshwater	0 result
	Microcystis panniformis FACHB-1757	Freshwater	0 result
	Microcystis sp. MC19	Freshwater	0 result
	Microcystis viridis NIES-102	Freshwater	0 result
Synechocystis	*Synechocystis* sp. PCC 6803	Freshwater	3 results
	Synechocystis sp. PCC 6714	Freshwater	1 result
	Synechocystis sp. IPPAS B-1465	Freshwater	3 results
Cyanobacterium	*Cyanobacterium aponinum* PCC 10605	Thermal spring	7 results
	Cyanobacterium sp. HL-69	Hypersaline Lake	0 result
Halothece	*Halothece* sp. PCC 7418	Freshwater	1 result
Euhalothece	*Euhalothece natronophila* Z-M001	Lagoon	0 result
Atelocyanobacterium	*Candidatus Atelocyanobacterium thalassa*	Marine	0 result
Crocosphaera	*Crocosphaera subtropica* ATCC 51142	Marine	1 result
	Crocosphaera watsonii WH 8501	Marine	3 results
Rippkaea	*Rippkaea orientalis* PCC 8801	Freshwater	0 result
	Rippkaea *orientalis* PCC 8802	Freshwater	0 result
Gloeothece	*Gloeothece verrucosa* PCC 7822	Freshwater	1 result
Cyanothece	*Cyanothece* sp. PCC 7425	Freshwater	4 results
Trichodesmium	*Trichodesmium erythraeum* IMS101	Marine	0 result
Microcoleus	*Allocoleopsis franciscana* PCC 7113	Terrestrial	1 result
Arthrospira	*Arthrospira platensis* NIES-39	Freshwater	1 result
Oxynema	*Oxynema aestuarii* AP17	Terrestrial	1 result
Limnospira	*Limnospira fusiformis* SAG 85.79	Lagoon	0 result
Geitlerinema	*Geitlerinema* sp. PCC 7407	Unknown	0 result
Oscillatoria	*Oscillatoria acuminata* PCC 6304	Terrestrial	0 result

	Oscillatoria nigro-viridis PCC 7112	Terrestrial	0 result
Moorea	*Moorea producens* PAL-8-15-08-1	Marine	2 results
Crinalium	*Crinalium epipsammum* PCC 9333	Terrestrial	0 result
Gloeobacter	*Gloeobacter violaceus* PCC 7421	Terrestrial	5 results
	Gloeobacter kilaueensis JS1	Terrestrial	2 results
Nostoc	*Nostoc* sp. PCC 7120	Freshwater	2 results
	Nostoc punctiforme PCC 73102	Terrestrial	3 results
	Nostoc sp. PCC 7107	Freshwater	6 results
	Nostoc sp. PCC 7524	Thermal spring	12 results
	Nostoc sp. NIES-3756	Terrestrial	5 results
	Nostoc flagelliforme CCNUN1	Terrestrial	1 result
	Nostoc sp. CENA543	Lagoon	3 results
	Nostoc sphaeroides CCNUC1	Terrestrial	2 results
Trichormus	*Trichormus variabilis* ATCC 29413	Freshwater	3 results
	Nostoc azollae 0708	Freshwater	1 result
Anabaena	*Anabaena* sp. 90	Freshwater	0 result
	Anabaena cylindrica PCC 7122	Freshwater	2 results
	Anabaena sp. WA102	Freshwater	1 result
Cylindrospermum	*Cylindrospermum stagnale* PCC 7417	Terrestrial	1 result
Calothrix	*Calothrix* sp. PCC 6303	Freshwater	1 result
	Calothrix sp. 336/3	Freshwater	2 results
Fischerella	*Fischerella* sp. NIES-3754	Thermal spring	1 result
Nodularia	*Nodularia spumigena* UHCC 0039	Coastal	1 result
Dolichospermum	*Dolichospermum* sp. UHCC 0315A	Coastal	0 result
	Dolichospermum flos-aquae CCAP 1403/13F	Freshwater	0 result
Uncalssified Nostocales	*Nostocales* cyanobacterium HT-58-2	Terrestrial	6 results
Chroococcidiopsis	*Chroococcidiopsis thermalis* PCC 7203	Terrestrial	20 results
Pleurocapsa	*Pleurocapsa* sp. PCC 7327	Thermal spring	3 results
Stanieria	*Stanieria cyanosphaera* PCC 7437	Freshwater	3 results
	Stanieria sp. NIES-3757	Freshwater	3 results

Movie S1.

Chromatin three-dimensional conformation of *Synechocystis* sp. PCC 6803 wild-type strain under Fe-replete conditions.

Movie S2.

Chromatin three-dimensional conformation of *Synechocystis* sp. PCC 6803 wild-type strain under Fe-deplete conditions.

REFERENCES AND NOTES

1. W. Elbert, B. Weber, S. Burrows, J. Steinkamp, B. Büdel, M. O. Andreae, U. Pöschl, Contribution of cryptogamic covers to the global cycles of carbon and nitrogen. *Nat. Geosci.* **5**, 459–462 (2012).

2. P. Sánchez-Baracaldo, G. Bianchini, J. D. Wilson, A. H. Knoll, Cyanobacteria and biogeochemical cycles through Earth history. *Trends Microbiol.* **30**, 143–157 (2022).

3. P. Flombaum, J. L. Gallegos, R. A. Gordillo, J. Rincón, L. L. Zabala, N. Jiao, D. M. Karl, W. K. W. Li, M. W. Lomas, D. Veneziano, C. S. Vera, J. A. Vrugt, A. C. Martiny, Present and future global distributions of the marine Cyanobacteria *Prochlorococcus* and *Synechococcus. Proc. Natl. Acad. Sci. U.S.A.* **110**, 9824–9829 (2013).

4. T. Veaudor, V. Blanc-Garin, C. Chenebault, E. Diaz-Santos, J.-F. Sassi, C. Cassier-Chauvat, F. Chauvat, Recent advances in the photoautotrophic metabolism of Cyanobacteria: Biotechnological implications. *Life* **10**, 71 (2020).

5. R. R. Crichton, *Iron Metabolism: From Molecular Mechanisms to Clinical Consequences* (John Wiley and Sons, Ltd, ed. 4, 2016).

6. N. Keren, R. Aurora, H. B. Pakrasi, Critical roles of bacterioferritins in iron storage and proliferation of cyanobacteria. *Plant Physiol.* **135**, 1666–1673 (2004).

7. L. A. Finney, T. V. O’Halloran, Transition metal speciation in the cell: Insights from the chemistry of metal ion receptors. *Science* **300**, 931–936 (2003).

8. S. Shcolnick, T. C. Summerfield, L. Reytman, L. A. Sherman, N. Keren, The mechanism of iron homeostasis in the unicellular cyanobacterium *Synechocystis* sp. PCC 6803 and its relationship to oxidative stress. *Plant Physiol.* **150**, 2045–2056 (2009).

9. M. A. Saito, A. E. Noble, A. Tagliabue, T. J. Goepfert, C. H. Lamborg, W. J. Jenkins, Slow-spreading submarine ridges in the South Atlantic as a significant oceanic iron source. *Nat. Geosci.* **6**, 775–779 (2013).

10. M. J. A. Rijkenberg, R. Middag, P. Laan, L. J. A. Gerringa, H. M. van Aken, V. Schoemann, J. T. M. de Jong, H. J. W. de Baar, The distribution of dissolved iron in the west atlantic ocean. *PLOS ONE* **9**, e101323 (2014).

11. M. Hatta, C. I. Measures, J. Wu, S. Roshan, J. N. Fitzsimmons, P. Sedwicek, P. Morton, An overview of dissolved Fe and Mn distributions during the 2010–2011 U.S. GEOTRACES north Atlantic cruises: GEOTRACES GA03. *Deep Sea Res. II: Top. Stud. Oceanogr.* **116**, 117–129 (2015).

12. M. J. Behrenfeld, A. J. Bale, Confirmation of iron limitation of phytoplankton photosynthesis in the equatorial Pacific Ocean. *Nature* **383**, 508–511 (1996).

13. M. J. Behrenfeld, A. J. Milligan, Photophysiological expressions of iron stress in phytoplankton. *Ann. Rev. Mar. Sci.* **5**, 217–246 (2013).

14. Y. Shaked, H. Lis, Disassembling iron availability to phytoplankton. *Front. Microbiol.* **3**, doi.org/10.3389/fmicb.2012.00123 (2012).

15. H.-B. Jiang, W.-J. Lou, W.-T. Ke, W.-Y. Song, N. M. Price, B.-S. Qiu, New insights into iron acquisition by cyanobacteria: An essential role for ExbB-ExbD complex in inorganic iron uptake. *ISME J.* **9**, 297–309 (2015).

16. N. Xu, G.-W. Qiu, W.-J. Lou, Z.-K. Li, H.-B. Jiang, N. M. Price, B.-S. Qiu, Identification of an iron permease, cFTR1, in cyanobacteria involved in the iron reduction/re-oxidation uptake pathway. *Environ. Microbiol.* **18**, 5005–5017 (2016).

17. G. W. Qiu, W. J. Lou, C. Y. Sun, N. Yang, B. S. Qiu, Characterization of outer membrane iron uptake pathways in the model cyanobacterium *Synechocystis* sp. PCC 6803. *Appl. Environ. Microbiol.* **84**, e01512–e01518 (2018).

18. G. W. Qiu, C. Koedooder, B. S. Qiu, Y. Shaked, N. Keren, Iron transport in cyanobacteria - from molecules to communities. *Trends Microbiol.* **30**, 229–240 (2021).

19. C. W. Yong, B. Deng, L. M. Liu, X. W. Wang, H. B. Jiang, Diversity and evolution of iron uptake pathways in marine cyanobacteria from the perspective of the coastal strain *Synechococcus* sp. strain PCC 7002. *Appl. Environ. Microbiol.* **89**, e0173222 (2023).

20. A. K. Singh, L. A. Sherman, Reflections on the function of IsiA, a cyanobacterial stress-inducible, Chl-binding protein. *Photosynth Res.* **93**, 17–25 (2007).

21. G. Sandmann, R. Malkin, Iron-sulfur centers and activities of the photosynthetic electron transport chain in iron-deficient cultures of the Blue-Green Alga *Aphanocapsa*. *Plant Physiol.* **73**, 724–728 (1983).

22. L. M. Liu, D. L. Li, B. Deng, X. W. Wang, H. B. Jiang, Special roles for efflux systems in iron homeostasis of non-siderophore-producing cyanobacteria. *Environ. Microbiol.* **24**, 551–565 (2021).

23. C. Kranzler, M. Rudolf, N. Keren, E. Schleiff, Iron in Cyanobacteria *Adv. Bot. Res.***65**, 57–105(2013).

24. S. J. Dixon, B. R. Stockwell, The role of iron and reactive oxygen species in cell death. *Nat. Chem. Biol.* **10**, 9–17 (2014).

25. K. Hantke, Iron and metal regulation in bacteria. *Curr. Opin. Microbiol.* **4**, 172–177 (2001).

26. P. Cornelis, Q. Wei, S. C. Andrews, T. Vinckx, Iron homeostasis and management of oxidative stress response in bacteria. *Metallomics* **3**, 540–549 (2011).

27. M. Ghassemian, N. A. Straus, Fur regulates the expression of iron-stress genes in the cyanobacterium *Synechococcus* sp. strain PCC 7942. *Microbiology* **142**, 1469–1476 (1996).

28. J. A. Hernández, M. T. Bes, M. F. Fillat, J. L. Neira, M. L. Peleato, Biochemical analysis of the recombinant Fur (ferric uptake regulator) protein from *Anabaena* PCC 7119: Factors affecting its oligomerization state. *Biochem. J.* **366**, 315–322 (2002).

29. J. A. Hernández, S. López-Gomollón, M. T. Bes, M. F. Fillat, M. L. Peleato, Three fur homologues from Anabaena sp. PCC7120: Exploring reciprocal protein-promoter recognition. *FEMS Microbiol. Lett.* **236**, 275–282 (2004).

30. A. González, M. T. Bes, F. Barja, M. L. Peleato, M. F. Fillat, Overexpression of FurA in *Anabaena* sp. PCC 7120 reveals new targets for this regulator involved in photosynthesis, iron uptake and cellular morphology. *Plant Cell Physiol.* **51**, 1900–1914 (2010).

31. A. González, M. T. Bes, A. Valladares, M. L. Peleato, M. F. Fillat, FURA is the master regulator of iron homeostasis and modulates the expression of tetrapyrrole biosynthesis genes in *Anabaena* sp. PCC 7120. *Environ. Microbiol.* **14**, 3175–3187 (2012).

32. M. F. Fillat, The FUR (ferric uptake regulator) superfamily: Diversity and versatility of key transcriptional regulators. *Arch. Biochem. Biophys.* **546**, 41–52 (2014).

33. M. Ludwig, T. T. Chua, C. Y. Chew, D. A. Bryant, Fur-type transcriptional repressors and metal homeostasis in the cyanobacterium *Synechococcus* sp. PCC 7002. *Front. Microbiol.* **6**, 1217 (2015).

34. A. González, A. Valladares, M. L. Peleato, M. F. Fillat, FurA influences heterocyst differentiation in *Anabaena* sp. PCC 7120. *FEBS Lett.* **587**, 2682–2690 (2013).

35. S. López-Gomollón, E. Sevilla, M. T. Bes, M. L. Peleato, M. F. Fillat, New insights into the role of Fur proteins: FurB (*All2473*) from *Anabaena* protects DNA and increases cell survival under oxidative stress. *Biochem. J.* **418**, 201–207 (2009).

36. A. González, M. T. Bes, M. L. Peleato, M. F. Fillat, Expanding the role of FurA as essential global regulator in cyanobacteria. *PLOS ONE* **11**, e0151384 (2016).

37. L. Vuorijoki, A. Tiwari, P. Kallio, E.-M. Aro, Inactivation of iron-sulfur cluster biogenesis regulator SufR in *Synechocystis* sp. PCC 6803 induces unique iron-dependent protein-level responses. *Biochim. Biophys. Acta Gen. Subj.* **1861**, 1085–1098 (2017).

38. C. Dan, H. Qingfang, W. L. Araujo, PfsR is a key regulator of iron homeostasis in *Synechocystis* PCC 6803. *PLOS ONE* **9**, e101743 (2014).

39. M. A. Hernández-Prieto, V. Schön, J. Georg, L. Barreira, J. Varela, W. R. Hess, M. E. Futschik, Iron deprivation in *Synechocystis*: Inference of pathways, non-coding RNAs, and regulatory elements from comprehensive expression profiling. *G3: Genes, Genomes, Genet.* **2**, 1475–1495 (2012).

40. U. Dühring, I. M. Axmann, W. R. Hess, A. Wilde, An internal antisense RNA regulates expression of the photosynthesis gene *isiA*. *Proc. Natl. Acad. Sci. U.S.A.* **103**, 7054–7058 (2006).

41. J. Georg, G. Kostova, L. Vuorijoki, V. Schön, T. Kadowaki, T. Huokko, D. Baumgartner, M. Müller, S. Klähn, Y. Allahverdiyeva, Y. Hihara, M. E. Futschik, E.-M. Aro, W. R. Hess, Acclimation of oxygenic photosynthesis to iron starvation is controlled by the sRNA IsaR1. *Curr. Biol.* **27**, 1425–1436.e7 (2017).

42. José A. Hernández, A. M. Muro-Pastor, E. Flores, M. T. Bes, M. L. Peleato, María F. Fillat, Identification of a furA cis antisense RNA in the cyanobacterium *Anabaena* sp. PCC 7120. *J. Mol. Biol.* **355**, 325–334 (2006).

43. E. Sevilla, B. Martin-Luna, A. Gonzalez, J. A. Gonzalo-Asensio, M. L. Peleato, M. F. Fillat, Identification of three novel antisense RNAs in the fur locus from unicellular cyanobacteria. *Microbiology* **157**, 3398–404 (2011).

44. V. S. Lioy, A. Cournac, M. Marbouty, S. Duigou, J. Mozziconacci, O. Espéli, F. Boccard, R. Koszul, Multiscale structuring of the *E. coli* chromosome by nucleoid-associated and condensin proteins. *Cell* **172**, 771–783.e18 (2018).

45. X. Wang, H. B. Brandão, T. B. K. Le, M. T. Laub, D. Z. Rudner, *Bacillus subtilis* SMC complexes juxtapose chromosome arms as they travel from origin to terminus. *Science* **355**, 524–527 (2017).

46. T. B. K. Le, M. V. Imakaev, L. A. Mirny, M. T. Laub, High-resolution mapping of the spatial organization of a bacterial chromosome. *Science* **342**, 731–734 (2013).

47. K. Bhm, G. Giacomelli, A. Schmidt, A. Imhof, R. Koszul, M. Marbouty, M. Bramkamp, Chromosome organization by a conserved condensin-ParB system in the actinobacterium Corynebacterium glutamicum. *Nat. Commun.* **11**, 1485 (2020).

48. L. Deng, Z. Zhao, L. Liu, Z. Zhong, W. Xie, F. Zhou, W. Xu, Y. Zhang, Z. Deng, Y. Sun, Dissection of 3D chromosome organization in *Streptomyces coelicolor* A3(2) leads to biosynthetic gene cluster overexpression. *Proc. Natl. Acad. Sci. U.S.A.* **120**, e2222045120 (2023).

49. C.-T. Ong, V. G. Corces, CTCF: An architectural protein bridging genome topology and function. *Nat. Rev. Genet.* **15**, 234–246 (2014).

50. A. Miele, J. Dekker, Long-range chromosomal interactions and gene regulation. *Mol. Biosyst.* **4**, 1046–1057 (2008).

51. J. Dekker, Gene regulation in the third dimension. *Science* **319**, 1793–1794 (2008).

52. S. De, F. Michor, DNA secondary structures and epigenetic determinants of cancer genome evolution. *Nat. Struct. Mol. Biol.* **18**, 950–955 (2011).

53. T. Kaneko, S. Sato, H. Kotani, A. Tanaka, E. Asamizu, Y. Nakamura, N. Miyajima, M. Hirosawa, M. Sugiura, S. Sasamoto, T. Kimura, T. Hosouchi, A. Matsuno, A. Muraki, N. Nakazaki, K. Naruo, S. Okumura, S. Shimpo, C. Takeuchi, T. Wada, A. Watanabe, M. Yamada, M. Yasuda, S. Tabata, Sequence analysis of the genome of the unicellular Cyanobacterium *Synechocystis* sp. strain PCC6803. II. Sequence determination of the entire genome and assignment of potential protein-coding regions. *DNA Res.* **3**, 185–209 (1996).

54. E. P. Nora, B. R. Lajoie, E. G. Schulz, L. Giorgetti, I. Okamoto, N. Servant, T. Piolot, N. L. van Berkum, J. Meisig, J. Sedat, J. Gribnau, E. Barillot, N. Blüthgen, J. Dekker, E. Heard, Spatial partitioning of the regulatory landscape of the X-inactivation centre. *Nature* **485**, 381–385 (2012).

55. M. Trussart, E. Yus, S. Martinez, D. Baù, Y. O. Tahara, T. Pengo, M. Widjaja, S. Kretschmer, J. Swoger, S. Djordjevic, L. Turnbull, C. Whitchurch, M. Miyata, M. A. Marti-Renom, M. Lluch-

Senar, L. Serranoc, Defined chromosome structure in the genome-reduced bacterium Mycoplasma pneumoniae. *Nat. Commun.* **8**, 14665 (2017).

56. J. R. Dixon, S. Selvaraj, F. Yue, A. Kim, Y. Li, Y. Shen, M. Hu, J. S. Liu, B. Ren, Topological domains in mammalian genomes identified by analysis of chromatin interactions. *Nature* **485**, 376–380 (2012).

57. F. Ma, X. Zhang, X. Zhu, T. Li, J. Zhan, H. Chen, C. He, Q. Wang, Dynamic changes of IsiA-containing complexes during long-term iron deficiency in *Synechocystis* sp. PCC 6803. *Mol. Plant* **10**, 143–154 (2017).

58. P. Cao, D. Cao, L. Si, X. Su, L. Tian, W. Chang, Z. Liu, X. Zhang, M. Li, Structural basis for energy and electron transfer of the photosystem I–IsiA–flavodoxin supercomplex. *Nat. Plants* **6**, 167–176 (2020).

59. Y. Cheng, T. Zhang, L. Wang, W. Chen, Transcriptome analysis reveals IsiA-regulatory mechanisms underlying iron depletion and oxidative-stress acclimation in *Synechocystis* sp. strain PCC 6803. *Appl. Environ. Microbiol.* **86**, e00517–20 (2020).

60. D. E. Heinrichs, K. Poole, Cloning and sequence analysis of a gene (pchR) encoding an AraC family activator of pyochelin and ferripyochelin receptor synthesis in *Pseudomonas aeruginosa*. *J. Bacteriol.* **175**, 5882–5889 (1993).

61. B. Soni, L. Houot, C. Cassier-Chauvat, F. Chauvat, Prominent role of the three *Synechocystis* PchR-like regulators in the defense against metal and oxidative stresses. *Open J. Biochem.* 1 (2012).

62. A. Nodop, D. Pietsch, R. Höcker, A. Becker, E. K. Pistorius, K. Forchhammer, K.-P. Michel, Transcript profiling reveals new insights into the acclimation of the mesophilic fresh-water cyanobacterium *Synechococcus elongatus* PCC 7942 to iron starvation. *Plant Physiol.* **147**, 747–763 (2008).

63. M. Ludwig, D. A. Bryant, Acclimation of the global transcriptome of the cyanobacterium *Synechococcus* sp. strain PCC 7002 to nutrient limitations and different nitrogen sources. *Front. Microbiol.* **3**, 145 (2012).

64. N. R. Cohen, W. Gong, D. M. Moran, M. R. McIlvin, M. A. Saito, A. Marchetti, Transcriptomic and proteomic responses of the oceanic diatom *Pseudo-nitzschia granii* to iron limitation. *Environ. Microbiol.* **20**, 3109–3126 (2018).

65. N. E. Gilbert, G. R. LeCleir, R. F. Strzepek, M. J. Ellwood, B. S. Twining, S. Roux, C. Pennacchio, P. W. Boyd, S. W. Wilhelm, Bioavailable iron titrations reveal oceanic *Synechococcus* ecotypes optimized for different iron availabilities. *ISME Commun.* **2**, 54 (2022).

66. R. T. Dame, F.-Z. M. Rashid, D. C. Grainger, Chromosome organization in bacteria: Mechanistic insights into genome structure and function. *Nat. Rev. Genet.* **21**, 227–242 (2020).

67. M. A. Umbarger, E. Toro, M. A. Wright, G. J. Porreca, D. Baù, S.-H. Hong, M. J. Fero, L. J. Zhu, M. A. Marti-Renom, H. H. McAdams, L. Shapiro, J. Dekker, G. M. Church, The three-dimensional architecture of a bacterial genome and its alteration by genetic perturbation. *Mol. Cell* **44**, 252–264 (2011).

68. M. Marbouty, A. Le Gall, D. I. Cattoni, A. Cournac, A. Koh, J.-B. Fiche, J. Mozziconacci, H. Murray, R. Koszul, M. Nollmann, Condensin- and replication-mediated bacterial chromosome folding and origin condensation revealed by Hi-C and super-resolution imaging. *Mol. Cell* **59**, 588–602 (2015).

69. A. González, M. T. Bes, M. L. Peleato, M. F. Fillat, Unravelling the regulatory function of FurA in Anabaena sp. PCC 7120 through 2-D DIGE proteomic analysis. *J. Proteomics* **74**, 660–671 (2011).

70. S. C. Andrews, A. K. Robinson, F. Rodríguez-Quiñones, Bacterial iron homeostasis. *FEMS Microbiol. Rev.* **27**, 215–237 (2003).

71. J.-W. Lee, J. D. Helmann, Functional specialization within the Fur family of metalloregulators. *Biometals* **20**, 485–499 (2007).

72. H.-B. Jiang, W.-J. Lou, H.-Y. Du, N. M. Price, B.-S. Qiu, Sll1263, a unique cation diffusion facilitator protein that promotes iron uptake in the cyanobacterium *Synechocystis* sp. strain PCC 6803. *Plant Cell Physiol.* **53**, 1404–1417 (2012).

73. H. K. Lichtenthaler, [34] Chlorophylls and carotenoids: Pigments of photosynthetic biomembranes. *Methods Enzymol.* **148**, 350–382 (1987).

74. D. Campbell, V. Hurry, A. K. Clarke, P. Gustafsson, G. Öquist, Chlorophyll fluorescence analysis of cyanobacterial photosynthesis and acclimation. *Microbiol. Mol. Biol. Rev.* **62**, 667–683 (1998).

75. M. Imakaev, G. Fudenberg, R. P. McCord, N. Naumova, A. Goloborodko, B. R. Lajoie, J. Dekker, L. A. Mirny, Iterative correction of Hi-C data reveals hallmarks of chromosome organization. *Nat. Methods* **9**, 999–1003 (2012).

76. E. Crane, Q. Bian, R. P. McCord, B. R. Lajoie, B. S. Wheeler, E. J. Ralston, S. Uzawa, J. Dekker, B. J. Meyer, Condensin-driven remodelling of X chromosome topology during dosage compensation. *Nature* **523**, 240–244 (2015).

77. F. Ay, T. L. Bailey, W. S. Noble, Statistical confidence estimation for Hi-C data reveals regulatory chromatin contacts. *Genome Res.* **24**, 999–1011 (2014).

78. N. Varoquaux, F. Ay, W. S. Noble, J.-P. Vert, A statistical approach for inferring the 3D structure of the genome. *Bioinformatics* **30**, i26–33 (2014).

79. J. Elhai, C. P. Wolk, A versatile class of positive-selection vectors based on the nonviability of palindrome-containing plasmids that allows cloning into long polylinkers. *Gene* **68**, 119–138 (1988).

80. T. A. Black, Y. Cai, C. P. Wolk, Spatial expression and autoregulation of hetR, a gene involved in the control of heterocyst development in Anabaena. *Mol. Microbiol.* **9**, 77–84 (1993).
